# Introducing Beneficial Alleles from Plant Genetic Resources into the Wheat Germplasm

**DOI:** 10.3390/biology10100982

**Published:** 2021-09-29

**Authors:** Shivali Sharma, Albert W. Schulthess, Filippo M. Bassi, Ekaterina D. Badaeva, Kerstin Neumann, Andreas Graner, Hakan Özkan, Peter Werner, Helmut Knüpffer, Benjamin Kilian

**Affiliations:** 1 Global Crop Diversity Trust, Platz der Vereinten Nationen 7, D-53113 Bonn, Germany; shivalipbg@gmail.com (S.S.); peter44429@gmail.com (P.W.); 2 Leibniz Institute of Plant Genetics and Crop Plant Research (IPK), OT Gatersleben, Corrensstr. 3, D-06466 Seeland, Germany; schulthess@ipk-gatersleben.de (A.W.S.); neumannk@ipk-gatersleben.de (K.N.); graner@ipk-gatersleben.de (A.G.); HKnuepffer@web.de (H.K.); 3 International Center for Agricultural Research in the Dry Areas (ICARDA), Rabat 10112, Morocco; f.bassi@cgiar.org; 4 N.I. Vavilov Institute of General Genetics, Russian Academy of Sciences, 119991 Moscow, Russia; badaeva@vigg.ru; 5 The Federal Research Center Institute of Cytology and Genetics, Siberian Branch of the Russian Academy of Sciences (ICG SB RAS), 630090 Novosibirsk, Russia; 6 Department of Field Crops, Faculty of Agriculture, University of Çukurova, Adana 01330, Turkey; hozkan@cu.edu.tr

**Keywords:** crop wild relatives, pre-breeding, crop improvement, germplasm enhancement, *Aegilops*, *Triticum*, plant genetic resources, genebank

## Abstract

**Simple Summary:**

Many crops including wheat have a narrow genetic base after hundreds of years of breeding and selection. This makes it difficult to breed new varieties with increased yields to feed the growing global population, and with stronger tolerance to the wider range of biotic and abiotic stresses that are anticipated with climate change. Thus, there is a need to introduce new genetic diversity into wheat breeding programs. Plant genetic resources stored in genebanks and the wild relatives of crops are potential sources of new genetic diversity. Here, we discuss the importance of these resources for breeding new wheat cultivars, and outline where they are currently stored and used. We also discuss pre-breeding, where genetic regions associated with desirable traits are identified and transferred into materials ready for use in breeding programs. Pre-breeding in wheat, when conducted in close collaboration with breeders, farmers, and end-users, has contributed to many outstanding varieties and novel beneficial diversity. This review addresses various genetic and genomic considerations for the strategic transfer of this useful diversity.

**Abstract:**

Wheat (*Triticum* sp.) is one of the world’s most important crops, and constantly increasing its productivity is crucial to the livelihoods of millions of people. However, more than a century of intensive breeding and selection processes have eroded genetic diversity in the elite genepool, making new genetic gains difficult. Therefore, the need to introduce novel genetic diversity into modern wheat has become increasingly important. This review provides an overview of the plant genetic resources (PGR) available for wheat. We describe the most important taxonomic and phylogenetic relationships of these PGR to guide their use in wheat breeding. In addition, we present the status of the use of some of these resources in wheat breeding programs. We propose several introgression schemes that allow the transfer of qualitative and quantitative alleles from PGR into elite germplasm. With this in mind, we propose the use of a stage-gate approach to align the pre-breeding with main breeding programs to meet the needs of breeders, farmers, and end-users. Overall, this review provides a clear starting point to guide the introgression of useful alleles over the next decade.

## 1. Introduction

Wheat (*Triticum* sp.) is one of the most widely grown food grain crops, feeding about 35% of the world’s population [1]. The global production of wheat is about 766 million tons, and it is cultivated across nearly 216 million hectares in more than 125 countries. Asia is the largest wheat producer, followed by Europe, the Americas, Oceania, and Africa [2]. More than 50% of the global wheat crop is produced by five countries: China, India, the Russian Federation, the USA, and France. Wheat productivity is highest in Europe (4.2 t ha^−1^), due to favorable natural conditions and intensive and innovative production systems, followed by Asia (3.4 t ha^−1^) [3].

Currently, five domesticated *Triticum* taxa are grown on a larger scale: (i) diploid *T. monococcum* (Scientific plant names are given without author in the text. For more information see Section 3 below) (einkorn wheat, 2n = 2x = 14, A^b^ genome); (ii) tetraploid *T. dicoccon* (emmer wheat, 2n = 4x = 28, BBAA) and *T. durum* (durum wheat, 2n = 4x = 28, BBAA); and (iii) hexaploid *T. aestivum* (bread wheat, 2n = 6x = 42, BBAADD) and *T. spelta* (dinkel wheat, 2n = 6x = 42, BBAADD) [4]. However, modern wheat production is primarily based on bread wheat, also known as common or soft wheat, and durum or macaroni wheat, accounting for 90–95% and 5–10% of global wheat production, respectively [5,6]. Bread wheat is mainly used as flour for various flatbreads, sourdough breads, and other baked goods. Durum wheat is mainly used to make semolina for pasta, couscous, and several baked goods, or as grains for bulgur. The other three domesticated wheats mentioned above are grown on a much smaller scale, mainly for organic or niche foods [6,7,8,9].

Since the “Green Revolution”, global wheat production (222.4 million tons in 1961 and 765.8 million tons in 2019) and productivity (1088.9 kg ha^−1^ in 1961 to 3546.8 kg ha^−1^ in 2019) have tripled, while the wheat growing area (204 million ha in 1961 and 215.8 million ha in 2019) has remained the same [2]. These increases are due to the contribution of better agronomic managements in combination with the efforts by breeding programs to improve the genetic potential of cultivars in the form of response to inputs, better biotic and abiotic stress tolerance/resistance, and more targeted phenology.

The concept of ‘genetic gain’ describes genetic improvement or breeding progress, and is measured by the difference between a selected population and its progeny population. The expected genetic gain per year can be defined as follows: Δ*G* = *i r* σ*_A_*/*t*, where Δ*G* is the response to selection, *i* is the selection intensity (mean deviation of selected individuals in units of phenotypic standard deviation), *r* is the selection accuracy, σ*_A_* is the standard deviation of breeding values [10], and *t* is the duration of the breeding cycle.

The average annual genetic gain of wheat is ~1% [11]. To meet the food demands of the growing global population, an increase by ~1.7% annually is needed to reach a production of about one billion tons in 2050 [12]. Genetic gain is a critical component of productivity increase, and it relies on the ability of breeders to deliver superior cultivars every year. All crops have shown positive trends in genetic gains since the Green Revolution (e.g., [13,14,15,16,17]). However, recent studies show that genetic gain has already plateaued in several countries, and it seems unlikely to achieve the same progress in the coming decades. Additionally, yields of major crops including wheat, rice, maize and soybean have stagnated or even collapsed due to harsher climatic conditions in some parts of the world [18,19,20]. Positive trends in genetic gain continue to be achieved in individual breeding programs, but at the expense of eroding large parts of genetic diversity [11,21,22,23]. Will it, therefore, be possible to increase or even maintain the rate of genetic gain in the coming decades, despite the deteriorating climate conditions?

To help breeders achieve this goal, researchers have proposed new or revised methods to improve selection accuracy, reduce the cycle time, and increase the selection intensity with limited resources [24,25,26]. However, these approaches are mostly based on the principle of “crossing the best with the best to get the best” [27], which has proven to be a reliable strategy for developing new cultivars. However, this approach tends to rapidly fix several genomic regions, thus substantially promoting the erosion of genetic diversity [28]. In turn, this influences the number of possible allelic combinations placed under selection, and hence reduces the “intensity of selection”, a critical factor for the genetic gain equation. This problem is exacerbated by worsening weather conditions causing the raise of stronger disease strains and adverse climatic conditions [29,30]. Recently, Miedaner and Juroszek [31] highlighted increasing disease risks especially for wheat rusts and *Fusarium* head blight (FHB) in northwestern Europe in the future. To meet these challenges, breeders have to continuously incorporate novel alleles from plant genetic resources (PGR) into the breeding programs [32,33,34,35,36,37,38,39,40].

In the past, the use of PGR, and especially crop wild relatives (CWR), was considered by breeders as a “last-option emergency solution” to address problems that could not be solved using the modern elite germplasm alone. This is because more time and resources are required to introgress desirable traits with minimal linkage drag into the cultivated background from unadapted germplasm, such as CWR or landrace materials, than from elite lines. A very good example of this reluctance is the resistance to FHB provided by the Chinese spring landrace ‘Sumai-3’. This landrace and its transferred resistance have been associated with poor agronomic performance [41,42], leading breeders to avoid its use. Nevertheless, ‘Sumai-3’ has been used widely in North America, where FHB is more extreme, and this has resulted in breeding more than 20 wheat lines including several leading cultivars in the US and Canada [43].

Breeders need to permanently deliver new and better varieties in the shortest possible time, which makes it difficult to engage in the long, laborious, and costly process of introgressing useful alleles from PGR. However, the importance of using novel alleles in breeding programs is now widely recognized, and many programs have begun employing a “pre-breeder” to transfer useful PGR diversity into elite germplasm that can be readily used as parents by the breeder [44,45,46,47]. A pre-breeder thus acts as a link between genebanks and breeding programs and strengthens the pipeline for variety development. Breeders, after several years of pre-breeding work, realize that some of the ‘novel’ CWR-derived germplasm lines can compete well with those developed through classical breeding [44,48,49]. An example is the wheat research program of the Consultative Group on International Agricultural Research (CGIAR), which used *Aegilops tauschii*, the wheat D genome donor, to produce cultivars that are now grown in 10% and 34% respectively, of the wheat cultivation area in India and China [50]. Similarly, the International Center for Agricultural Research in the Dry Areas (ICARDA)’s durum wheat program has released more than 125 cultivars in 22 countries, 38% of which included PGR in their development [11]. The most surprising finding is that a smaller number of successful hybridizations between PGR and modern cultivars made by pre-breeders can compete with breeders’ elite × elite germplasm obtained by carrying out hundreds of crosses each year.

Experts’ opinions still differ on the best way to exploit PGR in breeding. Most emphasize the need for clear trait prioritization and the use of well-characterized PGR for germplasm enhancement [51,52,53,54], while others advocate the use of PGR without prior information [11,45,55].

In the present review, we discuss the importance of PGR for wheat improvement, the current status of PGR use in wheat breeding programs, and propose a way forward for the efficient and effective use of PGR based on the needs of breeders and end-users.

## 2. Status of the Wheat Germplasm Conserved Ex Situ

For wheat improvement, extensive genetic diversity comprising advanced cultivars, breeding lines, traditional cultivars and landraces, genetic stocks, introgression lines, mutants, and CWR is conserved ex situ in genebanks worldwide. According to the Food and Agriculture Organization (FAO) World Information and Early Warning System (WIEWS) [56], nearly 855,000 accessions of *Triticum* are conserved in 218 genebanks located in 88 countries around the globe. An earlier overview based on a larger number of information sources [57] reported 727,000 *Triticum* accessions in 223 genebanks worldwide (Table 1). These collections differ in the amount and types of germplasm conserved. The largest wheat collection with ca. 111,700 accessions is held by the International Maize and Wheat Improvement Center (CIMMYT) genebank, followed by more than 57,000 accessions at the National Small Grains Germplasm Research Facility, United States Department of Agriculture-Agricultural Research Service, and ca. 37,800 accessions at ICARDA, at that time located in Syria [57]. About 67% of the total wheat germplasm conserved in ex situ genebanks is held in 20 genebanks [57], with each genebank housing over 10,000 accessions. About 127 genebanks hold small collections of fewer than 1000 accessions each. Most of the wheat germplasm conserved in genebanks is landraces, while only small proportions are CWR and genetic stocks. In addition to *Triticum*, about 42,300 accessions of *Aegilops* are held in 63 genebanks in 40 countries worldwide [56,57]. The genebanks with the largest collections of *Triticum* and *Aegilops* are listed in Table 1. Although wheat CWR constitute only a relatively small proportion (~3%) of wheat PGR in genebanks, they are valuable sources of genes [58,59,60]. Five wheat CWR taxa have been classified as underrepresented in genebanks and are considered medium or high priority for conservation [61].

Access to the germplasm conserved in genebanks and to the associated data is very important to enhance the use of germplasm in crop improvement programs. The Global Information System (http://www.fao.org/plant-treaty/areas-of-work/global-information-system accessed on 21 September 2021), which was developed by integrating and augmenting existing systems, serves as a global entry point to facilitate the exchange of information related to the conservation, management, and use of plant genetic resources for food and agriculture (PGRFA). Shaw et al. [62] advocated three major components of data management for handling PGR collections and their associated data. The first component enables genebanks to manage information on the germplasm collections including passport data, phenotypic data, seed stock regeneration, and requests for germplasm. Many genebanks have developed their own custom-made systems; however, GRIN-Global (https://www.grin-global.org/ accessed on 23 August 2021), which provides a standardized set of tools for managing genebank collections, is increasingly being used by many crop genebanks. The second component includes platforms to integrate information on accessions across collections. This allows plant breeders and scientists to explore the internationally available germplasm of their target species. Summaries of genebank holdings are held by the FAO for the purpose of global monitoring of PGR activities. Such platforms include EURISCO [63] (http://eurisco.ecpgr.org accessed on 21 September 2021) and Genesys (https://www.genesys-pgr.org accessed on 21 September 2021), which provide information on passport data and, where available, phenotypic data from a wide range of national and international plant germplasm collections. The third component is platforms that integrate genomic and phenomic data with associated passport data. Together, these components provide query, browsing, and visualization tools that allow users to explore the increasingly large and complex germplasm characterization data sets generated by high-throughput omics technologies.

Such platforms include Germinate [64,65] (https://ics.hutton.ac.uk/get-germinate accessed on 21 September 2021), Legume Information system [66] (https://legumeinfo.org accessed on 21 September 2021), and BRIDGE [67], which provide access to detailed experimental and trial data for subsets of germplasm that may or may not be held within the genebank system [64]. In addition, supporting organizations (institutions, universities, and private companies) have germplasm information that may be publicly available, even if it is not included in the core platforms.

## 3. Wheat Taxonomy, Domestication, and Genepool

### 3.1. Taxonomical Treatment of Triticum and Aegilops Taxa

Wheat belongs to the family *Poaceae*, subfamily *Pooideae,* tribe *Triticeae,* and the genus *Triticum* L. Several classification schemes have been proposed for wheat, e.g., based on morphological, cytogenetic, and genomic characteristics [68,69,70,71,72] (Table 2). At present, most ex situ genebanks use the classifications proposed by Dorofeev et al. [68] and van Slageren [72]; cf. Table 2. In this article, we largely follow Dorofeev et al. [68]. It is important to note that only four wild *Triticum* species have been identified to date: diploid *T. urartu* and *T. boeoticum*, and tetraploid *T. dicoccoides* and *T. araraticum*. Authors of scientific names in *Triticum* and *Aegilops* are given in Table 2 and Table 3. No wild hexaploid *Triticum* species is known (Table 2).

The genus *Aegilops* L. is most closely related to *Triticum* and comprises 23 species with three ploidy levels [60] (Table 3 and Table 4). Because of the genetic sister-group relationship between *Aegilops* and *Triticum*, some authors have proposed to merge them into one common genus, *Triticum* [58,73,74]. However, this idea is not supported by most taxonomists [60,72,75,76,77,78,79,80,81]. Table 3 gives an overview of the classification systems of *Aegilops*. Van Slageren [72] and Kilian et al. [60] distinguish 11 diploid and 12 polyploid species. Seven distinct genomes have been identified in diploid *Aegilops* species [79,82,83], and all of them, except for the T genome of *Ae. mutica*, are also present in polyploid *Aegilops* species (Table 4). However, a recent study based on Diversity Arrays Technology (DArT) markers [84] suggested that *Ae. neglecta* and *Ae. columnaris* may contain a modified version of the T (*Ae. mutica*) or S genome (*Ae. speltoides*), and that their genome formulae should therefore be changed to UUT^s^T^s^ (Table 4).

**Table 2:** Overview of selected wheat classifications. Botanical author abbreviations are according to the International Plant Names Index (IPNI; https://www.ipni.org accessed on 21 September 2021). * cf. Hammer et al. [69].

Ploidy Level	Common Name	Biological Status	Kernel Coverage	Genome Formula (Haploid) Considered in This Review	Taxon Name Considered in This Review	van Slageren [72]	Mac Key [70]	Dorofeev et al. [68]	Schiemann [71]
**2n = 2x = 14**									
	Urartu wheat, wild Urartu einkorn	Wild	Hulled	A^u^	*T. urartu* Thumanjan ex Gandil.	*T. urartu* Thumanjan ex Gandil.	*T. urartu* Thumanjan ex Gandil.	*T. urartu* Thumanjan ex Gandil.	
	Wild einkorn	Wild	Hulled	A^b^	*T*. *boeoticum* Boiss.	*T. monococcum* L. subsp. *aegilopoides* (Link) Thell.	*T*. *monococcum* L. subsp. *boeoticum* (Boiss.) Á. Löve et D. Löve	*T*. *boeoticum* Boiss.	*T*. *boeoticum* Boiss. em. Schiem.
	Einkorn, domesticated einkorn, small spelt	Domesticated	Hulled	A^b^	*T*. *monococcum* L.	*T. monococcum* L. subsp. *monococcum*	*T*. *monococcum* L. subsp. *monococcum*	*T*. *monococcum* L.	*T*. *monococcum* L.
	Sinskaya’s wheat	Domesticated	Free-threshing	A^b^	*T*. *sinskajae* A. Filat. et Kurkiev			*T*. *sinskajae* A. Filat. et Kurkiev	
**2n = 4x = 28**									
	Wild emmer	Wild	Hulled	BA	*T*. *dicoccoides* (Körn. ex Asch. et Graebn.) Schweinf.	*T. turgidum* L. subsp. *dicoccoides* (Körn. ex Asch. et Graebn.) Thell.	*T*. *turgidum* subsp. *dicoccoides* (Körn. ex Asch. et Graebn.) Thell.	*T*. *dicoccoides* (Körn. ex Asch. et Graebn.) Schweinf.	*T*. *dicoccoides* Körn.
	Emmer	Domesticated	Hulled	BA	*T*. *dicoccon* Schrank *	*T. turgidum* L. subsp. *dicoccum* (Schrank ex Schübl.) Thell.	*T. turgidum* subsp. *dicoccum* (Schrank ex Schübl.) Thell.	*T*. *dicoccum* Schrank ex Schübl.	*T*. *dicoccum* Schübl.
	Persian wheat, dika	Domesticated	Free-threshing	BA	*T*. *carthlicum* Nevski	*T. turgidum* L. subsp. *carthlicum* (Nevski) Á. Löve et D. Löve	*T*. *turgidum* subsp. *carthlicum* (Nevski) Á. Löve et D. Löve	*T*. *carthlicum* Nevski	*T*. *carthlicum* Nevski
	Durum wheat, macaroni wheat	Domesticated	Free-threshing	BA	*T*. *durum* Desf.	*T. turgidum* L. subsp. *durum* (Desf.) Husn.	*T*. *turgidum* subsp. *turgidum* convar. *durum* (Desf.) Mac Key	*T*. *durum* Desf.	*T*. *durum* Desf.
	Polish wheat	Domesticated	Free-threshing	BA	*T*. *polonicum* L.	*T. turgidum* L. subsp. *polonicum* (L.) Thell.	*T*. *turgidum* subsp. *turgidum* convar. *polonicum* (L.) Mac Key	*T*. *polonicum* L.	*T*. *polonicum* L.
	Khorasan wheat, Turanian wheat	Domesticated	Free-threshing	BA	*T*. *turanicum* Jakubz.	*T. turgidum* L. subsp. *turanicum* (Jakubz.) Á. Löve et D. Löve	*T*. *turgidum* subsp. *turgidum* convar. *turancium* (Jakubz.) Mac Key	*T*. *turanicum* Jakubz.	*T*. *orientale* Perciv.
	Rivet, cone, English wheat, turgid wheat, poulard wheat	Domesticated	Free-threshing	BA	*T*. *turgidum* L.	*T. turgidum* L. subsp. *turgidum*	*T*. *turgidum* L. subsp. *turgidum* convar. *turgidum*	*T*. *turgidum* L.	*T*. *turgidum* L.
	Georgian wheat, Colchic emmer, Karamyschev’s wheat	Domesticated	Hulled	BA	*T. karamyschevii* Nevski	*T. turgidum* L. subsp. *palaeocolchicum* Á. Löve et D. Löve	*T*. *turgidum* subsp. *georgicum* (Dekapr. et Menabde) Mac Key	*T. karamyschevii* Nevski	
	Ethiopian wheat	Domesticated	Free-threshing	BA	*T*. *aethiopicum* Jakubz.			*T*. *aethiopicum* Jakubz.	
	Espahanian wheat, Isfahanian emmer	Domesticated	Hulled	BA	*T*. *ispahanicum* Heslot	*T*. *ispahanicum* Heslot		*T*. *ispahanicum* Heslot	
	Jakubziner’s wheat	Domesticated	Free-threshing	BA	*T*. *jakubzineri* (Udachin et Schachm.) Udachin et Schachm.			*T*. *jakubzineri* (Udachin et Schachm.) Udachin et Schachm.	
	Araratian wild emmer, Armenian wild emmer	Wild	Hulled	GA^t^	*T*. *araraticum* Jakubz.	*T. timopheevii* subsp. *armeniacum* (Jakubz.) Mac Key ex van Slageren	*T*. *timopheevii* subsp. *armeniacum* (Jakubz.) Mac Key	*T*. *araraticum* Jakubz.	
	Militina’s wheat	Domesticated	Free-threshing	GA^t^	*T*. *militinae* Zhuk. et Migush.			*T*. *militinae* Zhuk. et Migush.	
	Timofeev’s wheat	Domesticated	Hulled	GA^t^	*T*. *timopheevii* (Zhuk.) Zhuk.	*T. timopheevii* (Zhuk.) Zhuk. subsp. *timopheevii*	*T*. *timopheevii* (Zhuk.) Zhuk. subsp. *timopheevii*	*T*. *timopheevii* (Zhuk.) Zhuk.	*T*. *timopheevii* Zhuk.
2n = 6x = 42									
	Common wheat, bread wheat	Domesticated	Free-threshing	BAD	*T*. *aestivum* L.	*T. aestivum* L. subsp. *aestivum*	*T*. *aestivum* L. subsp. *aestivum*	*T*. *aestivum* L.	*T*. *aestivum* L.
	Club wheat	Domesticated	Free-threshing	BAD	*T*. *compactum* Host	*T. aestivum* subsp. *compactum* (Host) Mac Key	*T*. *aestivum* subsp. *compactum* (Host) Mac Key	*T*. *compactum* Host	*T*. *compactum* Host
	Macha wheat	Domesticated	Hulled	BAD	*T*. *macha* Dekapr. et Menabde	*T. aestivum* subsp. *macha* (Dekapr. et Menabde) Mac Key	*T*. *aestivum* subsp. *macha* (Dekapr. et Menabde) Mac Key	*T*. *macha* Dekapr. et Menabde	*T*. *macha* Dekapr. et Menabde
	Petropavlovsky’s wheat	Domesticated	Free-threshing	BAD	*T*. *petropavlovskyi* Udachin et Migush.			*T*. *petropavlovskyi* Udachin et Migush.	
	Spelt wheat	Domesticated	Hulled	BAD	*T*. *spelta* L.	*T. aestivum* subsp. *spelta* (L.) Thell.	*T*. *aestivum* subsp. *spelta* (L.) Thell.	*T*. *spelta* L.	*T*. *spelta* L.
	Indian dwarf wheat, shot wheat	Domesticated	Free-threshing	BAD	*T*. *sphaerococcum* Perciv.	*T. aestivum* subsp. *sphaerococcum* (Perciv.) Mac Key	*T*. *aestivum* subsp. *sphaerococcum* (Perciv.) Mac Key	*T*. *sphaerococcum* Perciv.	*T*. *sphaerococcum* Perciv.
	Vavilov’s wheat	Domesticated	Hulled	BAD	*T*. *vavilovii* (Thumanjan) Jakubz.	*T*. *vavilovii* Jakubz.		*T*. *vavilovii* (Thumanjan) Jakubz.	
	Zhukovsky’s wheat	Domesticated	Hulled	GA^t^A^b^	*T*. *zhukovskyi* Menabde et Ericzjan	*T*. *zhukovskyi* Menabde et Ericzjan	*T*. *zhukovskyi* Menabde et Ericzjan	*T*. *zhukovskyi* Menabde et Ericzjan	

**Table 3:** Overview of selected *Aegilops* classifications. Botanical author abbreviations are according to IPNI (https://www.ipni.org accessed on 21 September 2021).

	Kilian et al. [60] and This Review	van Slageren [72]	Kimber et Sears [74]	Whitcombe [80]	Hammer [77,78]	Chennaveerayah [75]	Kihara [79]	Eig [76]	Zhukovsky [81]
**Subgenus *Aegilops***									
**Section *Aegilops***									
1	*Ae*. *biuncialis* Vis.	*Ae*. *biuncialis* Vis.	*T*. *macrochaetum* (Shuttlew. et É. Huet ex Duval-Jouve) K. Richt.	*Ae*. *lorentii* Hochst.	*Ae*. *lorentii* Hochst.	*Ae*. *biuncialis* Vis.	*Ae*. *biuncialis* Vis.	*Ae*. *biuncialis* Vis.	*Ae*. *biuncialis* Vis.
2	*Ae*. *columnaris* Zhuk.	*Ae*. *columnaris* Zhuk.	*T*. *columnare* (Zhuk.) Ros. Morris et Sears	*Ae*. *columnaris* Zhuk.	*Ae*. *columnaris* Zhuk.	*Ae*. *columnaris* Zhuk.	*Ae*. *columnaris* Zhuk.	*Ae*. *columnaris* Zhuk.	*Ae*. *columnaris* Zhuk.
3	*Ae*. *geniculata* Roth	*Ae*. *geniculata* Roth	*T*. *ovatum* (L.) Raspail	*Ae*. *ovata* L.	*Ae*. *geniculata* Roth	*Ae*. *ovata* L.	*Ae*. *ovata* L.	*Ae*. *ovata* L.	*Ae*. *ovata* L.
	subsp. *geniculata*								
	subsp. *gibberosa* (Zhuk.) K. Hammer								
4	*Ae*. *kotschyi* Boiss.	*Ae*. *kotschyi* Boiss.	*T*. *kotschyi* (Boiss.) Bowden	*Ae*. *kotschyi* Boiss.	*Ae*. *kotschyi* Boiss.	*Ae*. *kotschyi* Boiss.	*Ae*. *kotschyi* Boiss.	*Ae*. *kotschyi* Boiss.	
5	*Ae*. *neglecta* Req. ex Bertol.	*Ae*. *neglecta* Req. ex Bertol. (4x and 6x)	*T*. *triaristatum* (Willd.) Godr. et Gren. (4x and 6x)	*Ae*. *triaristata* Willd. (4x and 6x)	*Ae*. *neglecta* Req. ex Bertol.	*Ae*. *triaristata* Willd.	*Ae*. *triaristata* Willd. (4x and 6x)	*Ae*. *triaristata* Willd. (4x and 6x)	*Ae*. *triaristata* Willd. (4x and 6x)
	subsp. *neglecta* (4x)				subsp. *neglecta* (4x)				
	subsp. *recta* (Zhuk.) K. Hammer (6x)				subsp. *recta* (Zhuk.) K. Hammer (6x)				
6	*Ae*. *peregrina* (Hack.) Maire et Weiller	*Ae*. *peregrina* (Hack.) Maire et Weiller	*T*. *kotschyi* (Boiss.) Bowden	*Ae*. *peregrina* (Hack.) Maire et Weiller	*Ae*. *peregrina* (Hack.) Maire et Weiller	*Ae*. *variabilis* Eig	*Ae*. *variabilis* Eig	*Ae*. *variabilis* Eig	
	subsp. *peregrina*	var. *peregrina*			subsp. *peregrina*				
		var. *brachyathera* (Boiss.) Maire et Weiller			subsp. *cylindrostachys* (Eig et Feinbrun) Maire et Weiller				
7	*Ae*. *triuncialis* L.	*Ae*. *triuncialis* L.	*T*. *triunciale* (L.) Raspail	*Ae*. *triuncialis* L.	*Ae*. *triuncialis* L.	*Ae*. *triuncialis* L.	*Ae*. *triuncialis* L.	*Ae*. *triuncialis* L.	*Ae*. *triuncialis* L.
	subsp. *triuncialis*	var. *triuncialis*			subsp. *triuncialis*				
	subsp. *persica* (Boiss.) Zhuk.	var. *persica* (Boiss.) Eig			subsp. *persica* (Boiss.) Zhuk.	*Ae. persica* Boiss.			
8	*Ae*. *umbellulata* Zhuk.	*Ae*. *umbellulata* Zhuk.	*T*. *umbellulatum* (Zhuk.) Bowden	*Ae*. *umbellulata* Zhuk.	*Ae*. *umbellulata* Zhuk.	*Ae*. *umbellulata* Zhuk.	*Ae*. *umbellulata* Zhuk.	*Ae*. *umbellulata* Zhuk.	*Ae*. *umbellulata* Zhuk.
	subsp. *umbellulata*								
	subsp. *transcaucasica* Dorof. et Migush.				subsp. *transcaucasica* Dorof. et Migush.				
**Section *Comopyrum***									
9	*Ae*. *comosa* Sibth. et Sm.	*Ae*. *comosa* Sm. in Sibth. et Sm.	*T*. *comosum* (Sibth. et Sm.) K. Richt.	*Ae*. *comosa* Sibth. et Sm.	*Ae*. *comosa* Sibth. et Sm.	*Ae*. *comosa* Sibth. et Sm.	*Ae*. *comosa* Sibth. et Sm.	*Ae*. *comosa* Sibth. et Sm.	*Ae*. *comosa* Sibth. et Sm.
	subsp. *comosa*	var. *comosa*			subsp. *comosa*	subsp. *comosa*			
	subsp. *heldreichii* (Boiss.) Eig (syn.: var. *subventricosa* Boiss.)	var. *subventricosa* Boiss.			subsp. *heldreichii* (Boiss.) Eig	subsp. *heldreichii* (Boiss.) Eig		subsp. *heldreichii* (Holzm.) Eig	
10	*Ae*. *uniaristata* Vis.	*Ae*. *uniaristata* Vis.	*T*. *uniaristatum* (Vis.) K. Richt.	*Ae*. *uniaristata* Vis.	*Ae*. *uniaristata* Vis.	*Ae*. *uniaristata* Vis.	*Ae*. *uniaristata* Vis.	*Ae*. *uniaristata* Vis.	*Ae*. *uniaristata* Vis.
**Section *Cylindropyrum***									
11	*Ae*. *cylindrica* Host	*Ae*. *cylindrica* Host	*T*. *cylindricum* (Host) Ces., Pass. Et Gibelli	*Ae*. *cylindrica* Host	*Ae*. *cylindrica* Host	*Ae. cylindrica* Host	*Ae*. *cylindrica* Host	*Ae*. *cylindrica* Host	*Ae*. *cylindrica* Host
12	*Ae*. *markgrafii* (Greuter) K. Hammer	*Ae*. *caudata* L.	*T*. *dichasians* (Zhuk.) Bowden	*Ae*. *caudata* L.	*Ae*. *markgrafii* (Greuter) K. Hammer	*Ae. caudata* L.	*Ae*. *caudata* L.	*Ae*. *caudata* L.	*Ae*. *caudata* L.
**Section *Sitopsis***									
13	*Ae*. *bicornis* (Forssk.) Jaub. et Spach	*Ae*. *bicornis* (Forssk.) Jaub. et Spach	*T*. *bicorne* Forssk.	*Ae*. *bicornis* (Forssk.) Jaub. et Spach	*Ae*. *bicornis* (Forssk.) Jaub. et Spach	*T*. *bicorne* Forssk.	*Ae*. *bicornis* (Forssk.) Jaub. et Spach	*Ae*. *bicornis* (Forssk.) Jaub. et Spach	*Ae*. *bicornis* (Forssk.) Jaub. et Spach
		var. *bicornis*							
		var. *anathera* Eig							
14	*Ae*. *longissima* Schweinf. et Muschl.	*Ae*. *longissima* Schweinf. et Muschl.	*T*. *longissimum* (Schweinf. et Muschl.) Bowden	*Ae*. *longissima* Schweinf. et Muschl.	*Ae*. *longissima* Schweinf. et Muschl. emend. Eig s.l.	*T*. *longissimum* (Schweinf. et Muschl.) Bowden subsp. *longissimum*	*Ae*. *longissima* Schweinf. et Muschl.	*Ae*. *longissima* Schweinf. et Muschl.	*Ae*. *longissima* Schweinf. et Muschl. emend. Eig
					subsp. *longissima*				
15	*Ae*. *sharonensis* Eig	*Ae*. *sharonensis* Eig		*Ae*. *sharonensis* Eig	subsp. *sharonensis* (Eig) K. Hammer	subsp. *sharonensis* (Eig) Chennav.		*Ae*. *sharonensis* Eig	
16	*Ae*. *searsii* Feldman et Kislev ex K. Hammer	*Ae*. *searsii* Feldman et Kislev ex K. Hammer	*T*. *searsii* (Feldman et Kislev) Feldman	*Ae*. *searsii* Feldman et Kislev	*Ae*. *searsii* Feldman et Kislev ex K. Hammer				
17	*Ae*. *speltoides* Tausch	*Ae*. *speltoides* Tausch	*T*. *speltoides* (Tausch) Gren. ex K. Richt.	*Ae*. *speltoides* Tausch	*Ae*. *speltoides* Tausch	*T. speltoides* Tausch	*Ae*. *speltoides* Tausch	*Ae*. *speltoides* Tausch	*Ae*. *speltoides* Tausch
	subsp. *speltoides*	var. *speltoides*			subsp. *speltoides*	subsp. *aucheri* (Boiss.) Chennav.			
	subsp. *ligustica* (Savign.) Zhuk.	var. *ligustica* (Savign.) Fiori		*Ae*. *ligustica* (Savign.) Coss.	subsp. *ligustica* (Savign.) Zhuk.	subsp. *ligusticum* (Savign.) Chennav.		*Ae*. *ligustica* (Savign.) Coss.	
**Section *Vertebrata***									
18	*Ae*. *crassa* Boiss. (4x and 6x)	*Ae*. *crassa* Boiss. (4x and 6x)	*T*. *crassum* (Boiss.) Aitch. et Hemsl. (4x and 6x)	*Ae*. *crassa* Boiss. (4x and 6x)	*Ae. crassa* Boiss. (4x and 6x)	*Ae. crassa* Boiss.	*Ae*. *crassa* Boiss. (4x and 6x)	*Ae*. *crassa* Boiss. (4x and 6x)	*Ae*. *crassa* Boiss. (4x and 6x)
					subsp. *crassa*				
19	*Ae*. *vavilovii* (Zhuk.) Chennav. (6x)	*Ae*. *vavilovii* (Zhuk.) Chennav.	*T*. *syriacum* Bowden	*Ae*. *vavilovii* (Zhuk.) Chennav.	subsp. *vavilovii* Zhuk. (6×)	*Ae. vavilovii* (Zhuk.) Chennav.			
20	*Ae*. *juvenalis* (Thell.) Eig	*Ae*. *juvenalis* (Thell.) Eig	*T*. *juvenale* Thell.	*Ae*. *juvenalis* (Thell.) Eig	*Ae*. *juvenalis* (Thell.) Eig	*Ae. juvenalis* (Thell.) Eig	*Ae*. *juvenalis* (Thell.) Eig	*Ae*. *juvenalis* (Thell.) Eig	*Ae*. *turcomanica* Roshev.
					*Ae*. *turcomanica* Roshev.				
21	*Ae*. *tauschii* Coss.	*Ae*. *tauschii* Coss.	*T*. *tauschii* (Coss.) Schmalh.	*Ae*. *squarrosa* L.	*Ae*. *tauschii* Coss.	*Ae. squarrosa* L.	*Ae*. *squarrosa* L.	*Ae*. *squarrosa* L.	*Ae*. *squarrosa* L.
	subsp. *tauschii*					subsp. *squarrosa*			
	subsp. *strangulata* (Eig) Tzvelev								
22	*Ae*. *ventricosa* Tausch	*Ae*. *ventricosa* Tausch.	*T*. *ventricosum* Ces., Pass. et Gibelli	*Ae*. *ventricosa* Tausch	*Ae*. *ventricosa* Tausch	*Ae. ventricosa* Tausch	*Ae*. *ventricosa* Tausch	*Ae*. *ventricosa* Tausch	*Ae*. *ventricosa* Tausch
**Subgenus *Amblyopyrum***									
23	*Ae*. *mutica* Boiss.	*Amblyopyrum muticum* (Boiss.) Eig	*T*. *tripsacoides* (Jaub. et Spach) Bowden	*Ae*. *mutica* Boiss.	*Ae*. *mutica* Boiss.	*Amblyopyrum muticum* (Boiss.) Eig	*Ae*. *mutica* Boiss.	*Ae*. *mutica* Boiss.	*Ae*. *mutica* Boiss.
	subsp. *mutica*	var. *muticum*			var. *mutica*	subsp. *muticum*			
	subsp. *loliacea* (Jaub et Spach) Zhuk.	var. *loliaceum* (Jaub. et Spach) Eig			var. *loliacea* (Jaub. et Spach) Eig	subsp. *loliaceum* (Jaub. et Spach) Á. Löve			

**Table 4:** *Aegilops* species and their genomic formulae (per haploid genome) considered in this review (G). Genomic formulae of tetraploids and hexaploids are cited as “female × male parent.” Capital letters specify genome types, while superscripts indicate modifications.

Section/Subgenus	Diploid		Tetraploid		Hexaploid	
	Species	G	Species	G	Species	G
Section *Aegilops* L.	*Ae. umbellulata* Zhuk.	U	*Ae. biuncialis* Vis.	U^b^M^b^	*Ae. neglecta* subsp. *recta* (Zhuk.) K. Hammer	U^n^X^n^N^n^
			*Ae. columnaris* Zhuk.	U^c^X^c^		
			*Ae. geniculata* Roth	U^g^M^g^		
			*Ae. kotschyi* Boiss.	U^k^S^k^		
			*Ae. neglecta* Req. ex Bertol. subsp*. neglecta*	U^n^X^n^		
			*Ae. peregrina* (Hack.) Maire et Weiller	U^p^S^p^		
			*Ae. triuncialis* L.	U^t^C^t^		
Section *Comopyrum* (Jaub. et Spach) Zhuk.	*Ae. comosa* Sibth. et Sm.	M				
	*Ae. uniaristata* Vis.	N				
Section *Cylindropyrum* (Jaub. et Spach) Zhuk.	*Ae. markgrafii* (Greuter) K. Hammer	C	*Ae. cylindrica* Host	D^c^C^c^		
Section *Sitopsis* (Jaub. et Spach) Zhuk.	*Ae. bicornis* (Forssk.) Jaub. et Spach	S^b^				
	*Ae. longissima* Schweinf. et Muschl.	S^l^				
	*Ae. sharonensis* Eig	S^sh^				
	*Ae. searsii* Feldman et Kislev ex K. Hammer	S^s^				
	*Ae. speltoides* Tausch	S				
Section *Vertebrata* Zhuk. emend. Kihara	*Ae. tauschii* Coss.	D	*Ae. crassa* Boiss. subsp. *crassa* (4x)	D^1^X^cr^	*Ae. crassa* Boiss. subsp. *crassa* (6x)	D^1^D^2^X^cr^
			*Ae. ventricosa* Tausch	D^v^N^v^	*Ae. vavilovii* (Zhuk.) Chennav.	D^1^X^cr^S^v^
					*Ae. juvenalis* (Thell.) Eig	D^1^X^cr^U^j^
Subgenus *Amblyopyrum*	*Ae. mutica* Boiss.	T				

### 3.2. Evolution and Domestication History

The evolutionary and domestication history of wheat is complex, and only a few aspects are covered here. Important references are given for further details and in-depth studies. One of the most recent reviews is that of Zeibig et al. [85].

The mechanisms of the speciation and subsequent radiation of the *Triticum*-*Aegilops* species complex have been the subject of debate for more than a century. Based on comprehensive taxon sampling and genome sequencing of various cereal species, including CWR and key varieties, a complex process involving at least one homoploid hybrid speciation event as well as multiple rounds of introgression have been proposed as key events in the formation of the extant *Aegilops* and *Triticum* taxa [86,87,88,89,90,91].

Ancient hybridizations between *Triticum* and *Aegilops* species, followed by allopolyploidization, were key events in the evolution and domestication history of wheat [92,93,94]. According to comparative sequence analyses of the nuclear and chloroplast genomes, the *Triticum-Aegilops* species complex arose between 2.1 and 4.5 million years ago (MYA) [89,95,96]. The progenitor of the wheat B genome radiated from the ancestor of *Ae. speltoides* approximately 780,000–980,000 years ago [96], and the wild diploid wheat A genome donor *T. urartu* diverged from *T. boeoticum* about 550,000–760,000 years ago [96].

Independent hybridizations between *T. urartu* and an extinct or still unknown diploid species related to *Ae. speltoides* (SS genome) [89,97] led to the emergence of the two tetraploid species, *T. dicoccoides* (2n = 4x = 48, BBAA) and *T. araraticum* (2n = 4x = 28, GGA^t^A^t^) [89,95,98,99,100]. Of these two species, *T. dicoccoides* is considered to be the older species. According to various estimates, it may have originated between 0.7–0.8 MYA [90,101] and 0.4–0.5 MYA [92,95,102], while *T.* *araraticum* probably originated between 0.1–0.4 MYA [90,92,101].

Wild emmer wheat (*T. dicoccoides*) consists of two major lineages with distinct geographical origins: (i) the western or southern Levant group, and (ii) the central-eastern group [103,104,105,106]. The domestication history of emmer wheat is complex, and pre-domestication cultivation, hybridization between the two different lineages, and human migration have played important roles [103,106,107]. *Triticum dicoccoides* was among the first cereals domesticated in the Fertile Crescent; its domesticated form is known as *T*. *dicoccon* (2n = 4x = 28, BBAA). This domestication step was the key to the subsequent evolution of durum and bread wheat [108,109].

The origin of *T. durum* is still intensively debated. However, it probably originated as a result of two successful domestication events by ancient farmers: first, from wild emmer to domesticated emmer; and second, from cultivated, presumably naked forms of emmer to durum [110]. The Levant (Jordan, Lebanon, Israel, Palestine, and Syria) is considered to be the center of origin of durum wheat [4,111], which later spread along the same path as *T. dicoccon* [112,113]. Several authors suggested based on recent molecular data that *T. aethiopicum*, which is currently cultivated in Ethiopia, was potentially derived from a different domestication event [112,114,115,116], but this remains to be verified.

The tetraploid *T. araraticum,* the wild progenitor of the Timopheevii wheat lineage, potentially originated in Northern Iraq [101,117,118]. Based on analyses of nuclear and cytoplasmic genomes, *T. araraticum* can be divided into two subgroups: one subgroup (ARA-0) is widespread, while the other (ARA-1) is found only in South-eastern Turkey and North-western Syria [119]. *Triticum timopheevii* (2n = 4x = 28, GGA^t^A^t^), the domesticated form of *T. araraticum,* has been cultivated only in western Georgia in the recent past. The potential sister-group relationship between Timopheev’s wheat from Georgia (*T. timopheevii* *s*.*str*.) and the much more widespread prehistoric ‘New Glume Wheat’ (*T. timopheevii* *s*.*l*.), of which the oldest archaeobotanical records were found in Turkey, has been intensively discussed in Badaeva et al. [119].

The hexaploid *T. aestivum* emerged as a result of allopolyploidization, i.e., hybridization between a potentially domesticated tetraploid wheat belonging to the emmer lineage and the wild diploid *Ae. tauschii* [120,121,122]. The results of molecular [103,123,124] and cytogenetic [125,126] studies suggested that the wheat D genome was contributed by *Ae. tauschii* subsp. *strangulata*. According to molecular analyses and archaeobotanical findings, hexaploid bread wheat originated about 8,000 years ago [92,109,127,128] in the area of North-western Iran and the South-western Caspian Sea [103,124]. *Triticum aestivum* may have been the result of not just one, but a few hybridization events involving several *Ae*. *tauschii* genotypes and different tetraploid wheat parents [123].

The hexaploid *T. zhukovskyi* (GGA^t^A^t^A^m^A^m^) arose as a result of hybridization between the domesticated form of tetraploid wheat in the Timopheevi lineage—*T. timopheevii*, and domesticated einkorn, *T. monococcum* [129]. Interestingly, just one spike of *T. zhukovskyi* was discovered in the 1960s by Menabde and Ericzjan among spikes of *T. timopheevii* and *T. monococcum* harvested from one ‘Zanduri’ field in the Lechkhumi region of western Georgia [129,130,131]. The Zanduri spring wheat complex, which consisted of a mixture of *T*. *monococcum* and *T*. *timopheevii* landraces, was well adapted to Lechkhumi and Racha, two historical provinces of Georgia. Zanduri wheat showed remarkable resistance to fungal diseases (see Badaeva et al. [119] for more details on *T*. *timopheevii* and *T. zhukovskyi*). The seeds of this single *T. zhukovskyi* spike gave rise to 51 accessions that are now maintained in 18 genebanks worldwide, as documented by Genesys [132], while Knüpffer [57] reported 64 *T. zhukovskyi* accessions in 22 genebanks worldwide.

Perhaps the most important traits that were modified and selected during the domestication of wheat were the introduction of the free-threshing character and the removal of the brittle rachis character. Other characteristics that have been altered during domestication and subsequent breeding include seed size, plant height, grain hardness, number of tillers, seed dormancy, photoperiod sensitivity, and vernalization requirement [4,133,134,135,136].

### 3.3. The Wheat Genepool Concept

The *Triticum*-*Aegilops* species complex and related CWR harbor enormous genetic diversity for wheat improvement. Following the genepool concept of Harlan and de Wet [137], the *Triticum* and *Aegilops* species can be classified into three genepools based on crossability between cultivated and wild taxa. This provides a useful framework for the efficient use of PGR in wheat breeding programs.

According to [137], the primary genepool of bread wheat contains (i) freely crossable taxa such as cultivars and landraces of *T. aestivum*; (ii) the wheat A genome donor *T. urartu*; (iii) diploid wild *T. boeoticum* and domesticated *T*. *monococcum*; (iv) the wheat D genome donor *Ae. tauschii*; (v) wild emmer *T. dicoccoides*; and (vi) all domesticated BBAA taxa.

The secondary genepool comprises *Triticum* and *Aegilops* species that have at least one genome in common or partially in common with bread wheat. This genepool contains (i) taxa of the GGA^t^A^t^ lineage; and (ii) several *Aegilops* species, in particular, *Ae. speltoides*.

The tertiary genepool consists of more distantly related diploid and polyploid taxa with chromosomes that are not homologous to those of wheat (Table 4), and includes, for example, (i) *Aegilops* species including *Ae. geniculata* (U^g^U^g^M^g^M^g^), *Ae. cylindrica* (D^c^ D^c^C^c^C^c^)*, Ae. biuncialis* (U^b^U^b^M^b^M^b^), *Ae. triuncialis* (U^t^U^t^C^t^C^t^), *Ae. comosa* (MM), *Ae. markgrafii* (CC), *Ae. neglecta* subsp. *neglecta* (U^n^U^n^X^n^X^n^) and subsp. *recta* (U^n^U^n^X^n^X^n^N^n^N^n^), *Ae.* *peregrina* (U^p^U^p^S^p^S^p^), and *Ae. umbellulata* (UU); (ii) rye, *Secale cereale* L., and (iii) other CWR such as diploid (2n = 2x = 14, EE) or tetraploid (2n = 4x = 28, EEEE) *Thinopyrum elongatum* (Host) D.R. Dewey [=*Agropyron elongatum* (Host) P. Beauv.], *Thinopyrum ponticum* (Podp.) Z.-W. Liu et R.-C. Wang (2n = 10x = 70, EEEEEEE^St^E^St^E^St^E^St^), and *Dasypyrum villosum* (L.) Borbás (2n = 2x = 14, VV).

Many studies have explored the evolutionary and cytogenetic relationships between cultivated wheat and wild species, and have developed methods to transfer genes and genetic regions from primary, secondary, and tertiary gene pools into wheat cultivars [60,120,136,138,139,140,141,142,143,144,145,146].

## 4. Status of PGR Use for Wheat Improvement

In the beginning of the 20^th^ century, PGR were used to develop wheat cultivars with improved rust resistance, early flowering and maturity, and short stems [147]. A crossing program led by the Italian plant breeder Nazareno Strampelli used the Japanese cultivar ‘Akakomugi’ carrying the dwarfing gene *Rht8* and the early maturity gene *Ppd-D1* to develop notable wheat cultivars such as ‘Mentana’, ‘Ardito’, ‘Villa Glori’, and ‘Damiano’, which were widely grown in Argentina and China [147]. Later, the semidwarf wheat ‘Norin 10’ was developed, carrying *Rht1* and *Rht2* dwarfing genes originating from either of the Japanese landraces ‘Ojima-Wase’, ‘Shiro-Daruma’, or ‘Sōshū’; although the details are unknown due to the loss of records [148,149,150,151]. ‘Norin 10’ changed the face of wheat cultivation. It was used in crossing programs in Mexico to develop ‘photoperiod-insensitive’, high-yielding, semidwarf cultivars. Those cultivars, combined with the widespread availability of nitrogen fertilizer, triggered the Green Revolution in Mexico and Asia. Indeed, these wheat landraces possess many useful alleles for high 1000-kernel weight, plant biomass, and photosynthesis [152].

Many studies of landraces have their usefulness for various traits. For instance, Mexican wheat landraces have been reported to be highly adapted to temperature and drought stress [153,154], including the case of the cultivar ‘Aragon 03’ developed through selection from the landrace ‘Catalan de Monte’ [155]. Additionally, the very successful durum wheat variety ‘Senatore Cappelli’, bred by Nazareno Strampelli at the beginning of the past century and still cultivated today by organic farmers, traces its origin to the hybridization of two landraces [156].

PGR of other species have also been used widely. For instance, the tetraploid *T. polonicum* has longer glumes and grains, and has been used to breed wheat lines with increased grain size [157] or increased micronutrient contents [158]. It is likely that *T. polonicum* contributed these traits to *T. petropavlovskyi* [159,160] and the Portuguese landrace group ‘Arancada’ [161]. The tetraploid wheat *T. timopheevii* is strongly resistant to rust [68,162]. Two genes conferring resistance to leaf rust (*Lr18* and *Lr50*), three genes conferring resistance to stem rust (*Sr36*, *Sr37*, *Sr40*), and three genes conferring resistance to powdery mildew (*Pm6*, *Pm27*, *Pm37*) have been introgressed from *T. timopheevii* into bread wheat [163]. Besides resistance genes, cytoplasmic male sterility (CMS) induced by *T. timopheevii* cytoplasm offers great potential for hybrid heterosis in wheat [164,165,166].

Wheat genepools have contributed more than single genes to crop improvement efforts; entire chromosomal segments also have been introduced with noteworthy results. Perhaps the most important of these is the 1B/1R translocation that was identified as a simple transfer between rye and wheat in the cultivar ‘Kavkaz’, which was developed in the former Soviet Union. The 1B/1R translocation confers resistance to various diseases and adaptation to marginal environments [167]. This translocation has been deemed so important that it has been incorporated into several hundred wheat varieties [168,169,170,171,172], including the prominent ‘Veery’ lines that are grown across almost 50% of the wheat cultivation area in developing countries [173]. Useful alleles from polyploid wild species such as *Ae. ventricosa* (D^v^N^v^ genome) have been transferred into hexaploid wheat genotypes using a tetraploid wheat genotype (i.e., *T. carthlicum*, BBAA) as a bridge species. For example, the eyespot resistance gene *Pch1* was transferred from *Ae. ventricosa* (D^v^D^v^N^v^N^v^) line ‘AP-1’ into bread wheat cultivar ‘Almatense H-10-15’ using tetraploid wheat, *T. turgidum* (or *T. polonicum*) (BBAA) line ‘H-1-1’ as a bridge species [174]. The *Ae. ventricosa* 2N^v^S segment has been used extensively in wheat breeding programs worldwide to reduce lodging and improve disease resistance and crop yield [175,176,177,178,179].

Like other PGR, CWR of wheat carry novel alleles that control important traits [60,180,181]. CWR species are well adapted to biotic and abiotic stresses that are ubiquitous in their native distribution range, as well as to annual inter-climate variation. In the course of evolution, these species have accumulated a high diversity of alleles for stress tolerance and adaptation. Important genes identified or transferred from *Ae. tauschii* to wheat include those conferring resistance to diseases [rusts (leaf, stem and stripe), powdery mildew, *Septoria tritici, Septoria nodorum*, tan spot] and insect pests (cyst nematode, root knot nematode, Hessian fly, greenbug, Russian wheat aphid, wheat curl mite, and soil-borne cereal mosaic virus) [59,60,182,183,184].

However, the introgression of useful alleles from CWR into modern germplasm is often limited by cross-species incompatibility, the prevention of non-homologous recombination, and various problems related to cytoplasmic or meiotic sterility. Therefore, the successful production of a stable hybrid is a major achievement in itself. However, breaking of, or compensating for negative linkages of transferred CWR genomic regions has proven to be difficult [139]. Discovery of the *Ph1* locus led to the possibility of recombining non-homologous genomes with those of wheat. The same approach is now being followed in many other cereals [143,185,186,187] and has been extended to include a wider range of CWR in wheat [188]. Genomic analyses can reveal the translocations and rearrangements that have been introduced, allowing for more structured and efficient screening of the huge array of novel recombinations that can be generated. A single *Ph1-*deletion mutant, the *ph1b* mutant, has been used for the last 40 years to introgress important alleles from wild relatives into cultivated wheat lines through homoeologous pairing [187]. Furthermore, Chen et al. [189] transferred the *Ph1-*suppressor gene from *Ae. speltoides* into bread wheat. This bread wheat line is an efficient inducer of homoeologous pairing and has been used to transfer genes conferring leaf rust and stripe rust resistance from *Ae. umbellulata* [190], *Ae. triuncialis,* and *Ae. geniculata* [191] into various bread wheat lines.

Various studies have aimed to improve durum wheat and bread wheat by introgressing genes from other *Triticum* and *Aegilops* species by backcrossing and/or using synthetic hexaploids [192,193,194]. Durum wheat has been improved by crossing with *T. polonicum*, *T. carthlicum,* and *T. dicoccon* to increase its drought tolerance [195], and by crossing with *T. araraticum* [196] or *T. dicoccon* [197] to improve its resistance to Hessian fly. The development of synthetic hexaploids has provided a useful strategy for the efficient and enhanced use of diploid and tetraploid wild species for wheat improvement. Synthetic hexaploid wheat derived from crosses between *Ae. tauschii* or other *Aegilops* species and *T. monococcum*, *T. dicoccon,* or *T. durum* can be used to transfer useful genetic variation, including genetic regions related to adaptive traits, into modern bread wheat cultivars [198,199]. Synthetic hexaploid wheat obtained by crossing *Ae. tauschii* with *T. durum* often serves as a bridge to transfer useful traits into modern bread wheat cultivars. It has been reported that diploid *Ae. tauschii* can increase the grain weight and improve the grain yield of wheat, besides improving resistance to biotic stresses [200,201,202]. Synthetic wheat lines also exhibit excellent drought-adaptive traits, improved tolerance to heat, water logging, and freezing, and strong resistance to major diseases such as *Fusarium* head blight [203,204,205,206,207]. At present, synthetic hexaploid wheat lines are being deployed in breeding programs to broaden the genetic diversity of cultivated wheat lines [192,199]. The genetic contribution of *Ae. tauschii* to CIMMYT’s spring bread wheat improvement program through synthetic hexaploid wheat is well documented [198]. The use of synthetic wheat lines derived from crosses between *T*. *dicoccon* and *Ae. tauschii* has contributed several important genes conferring resistance to *Septoria nodorum* leaf blotch [208,209], Russian wheat aphid [210], and green bug [211].

With the recent advances in high-throughput screening technologies, King et al. [212,213] and Iefimenko et al. [214] developed introgressions from *Ae. mutica*, *Ae. speltoides,* and *Thinopyrum bessarabicum* (Savul. et Rayss) Á. Löve with an objective to introgress the entire genome of these species into wheat in small chromosome segments. A total of 66 stably inherited homozygous wheat/*Ae. mutica* introgression lines have been developed using a doubled-haploid procedure for use in wheat improvement [215].

Recent studies on the genomic and cytogenetic diversity, distribution, and domestication of the tetraploid GGA^t^A^t^ genepool will promote the introgression of useful variation from this hitherto neglected genepool into common wheat [119]. Other recent studies have explored the genomic diversity among 80,000 wheat accessions, including several lines from global breeding programs as well as old wheat cultivars. The results of those studies have revealed extensive structural rearrangements and identified several known and unknown introgressions [91,124,216]. Some introgressions were detected in wheat cultivars released in the first half of the 19th century, demonstrating that natural introgressions were used in early breeding history and still influence elite lines today [217]. Sansaloni et al. [216] and Kabbaj et al. [112] identified landraces with unexplored diversity. Such landraces can be used to introgress allelic diversity, which is lacking in current breeding programs, to develop the next generation of modern wheat varieties.

## 5. PGR of Known Value to Be Incorporated in the Future: Breeders’ Needs

Because of the narrow genetic base of modern wheat cultivars, germplasm enhancement in wheat is inadequate to achieve genetic gain. Different *Aegilops* species carry many useful characteristics such as resistance/tolerance to various biotic (diseases and insect pests) and abiotic (drought, salinity, extreme temperature, soil mineral toxicity, and deficiency) stresses, and traits related to high nutritional content and quality [44,59,60,85,141,180]. Due to the low genetic diversity in elite durum and bread wheat breeding programs, pre-breeding [44,52,218] may play an important role in creating novel genetic diversity using landraces and CWR as sources of genes and genetic regions conferring the traits outlined below.

### 5.1. Diversification of Resistance Genes

The emergence of new races of pathogens and the breakdown of wheat resistance loci are common, and have led to several epidemics in the past. In bread wheat and durum wheat, stem rust is one of the most devastating diseases. Recent epidemics in Ethiopia [219], Europe [220,221,222], and Central Asia [223] indicate that the disease is re-emerging as a threat to wheat production worldwide. High levels of resistance to virulent races, such as those in the Ug99 race group, are not available in the breeders’ working collections. Evaluation of *Aegilops* species from the tertiary genepool resulted in the identification of CWR with resistance to three highly virulent races of *Puccinia graminis* f. sp. *tritici*: TTKSK, TRTTF, and TTTTF [181]. Efforts are needed to transfer resistance genes from *Aegilops* species such as *Ae. biuncialis, Ae. markgrafii, Ae. comosa,* or *Ae. umbellulata* that do not share common genomes with cultivated wheat (Table 4). Recently, the breakdown of resistance to *Septoria tritici* blotch (STB; a disease caused by the fungal pathogen *Zymoseptoria tritici*) in the winter wheat cultivar ‘Cougar’ and its derivatives has been reported in the UK and Ireland [224]. These findings show that diverse sources of resistance need to be deployed in disease resistance breeding programs.

Barley yellow dwarf virus is one of the most serious viral pathogens of common wheat (*T. aestivum*) worldwide [225]. Resistance to the viral vectors, such as bird cherry-oat aphid (*Rhopalosiphum padi*) and English grain aphid (*Sitobion avenae*), has been identified in *T. boeoticum*, *Ae. tauschii*, *T. araraticum*, and *T. dicoccoides* [226].

### 5.2. Improved Tolerance to Drought, Heat, and Salinity

About 45% of wheat cultivated in developing countries is grown under rainfed conditions. Drought is one of the major production constraints in these regions, especially under changing climatic conditions [227,228,229]. Similarly, heat stress is projected to become a major threat to wheat production; a ~4–6% reduction in the average global yield of wheat is predicted for each ~1 °C increase in the global mean air temperature [29]. Salinity is another important limiting factor of wheat production worldwide. Einkorn and emmer wheats are better adapted to certain harsh environments. A possible approach is to use these wheats, adapted landraces, and CWR as donors in breeding programs [230,231,232]. An alternative approach would be to promote the cultivation of einkorn and emmer wheats in harsh environments, especially in developing countries, depending on the demand and climatic conditions [233,234,235]. *Triticum* *dicoccon*, *T. polonicum*, *T. carthlicum,* and *T. turanicum* are sources of useful alleles for improving abiotic and biotic stress tolerance in modern wheat cultivars [195,234,236,237].

### 5.3. Organic Farming

The intensive use of fertilizers and pesticides in wheat cultivation has raised concerns about biodiversity in general, and human and soil health in particular. Therefore, there is growing interest in low-input and organic farming. In the last decade, interest in einkorn (*T. monococcum*), emmer (*T. dicoccon*) and dinkel (*T. spelta*) has increased [235,238,239,240,241,242]. Due to their high nutritional value and their ability to grow in poor soils and under a range of climatic conditions, cultivation of these wheat species is expanding in Germany, Austria, Switzerland, Czech Republic, Italy, and Turkey. In particular, *T*. *spelta* has become increasingly popular in Europe and is widely cultivated, especially by organic farmers [243]. Emmer and einkorn wheats are less popular than spelt, although they are all considered “healthy cereals” and are recommended for people suffering from allergies, colitis, high cholesterol, and diabetes [244,245].

### 5.4. Improved Nutritional Quality

Compared with einkorn and emmer wheats, most of the durum and bread wheat cultivars have lower grain contents of minerals such as iron, zinc, phosphorus, magnesium, copper, manganese and selenium. Polish wheat (*T*. *polonicum*) contains higher concentrations of iron, zinc, magnesium, phosphorus, sulfur, and boron than do *T*. *durum* and *T*. *aestivum*, and can be used to improve the nutritional value of modern wheat cultivars [158]. *Triticum polonicum* may constitute valuable genetic material for breeding new wheat cultivars with a high nutritive value and satisfactory resistance to FHB [246]. Emmer wheat has a high fiber content, which makes it popular among consumers in Italy and Switzerland [247]. Similarly, compared with hard red wheats, Turkish cultivated einkorn (*T*. *monococcum*) landraces have higher protein content [248] and are consumed in the form of soups, salads, casseroles, and sauces [249]. It is important to encourage the cultivation and genetic improvement of diploid and tetraploid wheats, particularly *T*. *monococcum* and *T*. *dicoccon*, because of their high nutritional value and their importance in organic agriculture [234,235,250,251]. Wheat CWR hold great potential for biofortification. For instance, Çakmak et al. [252] screened many CWR accessions collected from the Fertile Crescent for their grain contents of iron, zinc, and other mineral nutrients. Wild emmer wheat, *T*. *dicoccoides* (825 accessions), showed wide variation and the highest concentrations of micronutrients, significantly exceeding those in cultivated wheat. The results indicated that wild emmer is an important genetic resource for improving grain quality and increasing the contents of mineral nutrients in modern cultivated wheats [253]. Compared with cultivated wheat, wild emmer wheat accumulates higher contents of iron and zinc [85,254], as do *T*. *boeoticum* and *T*. *monococcum* [255,256]. Among *Aegilops* species, *Ae*. *tauschii* and *Ae. kotschyi* show higher grain iron and zinc contents compared with cultivated wheat [257,258,259].

## 6. Exploitation of PGR in Breeding: A Stepwise Approach

**Figure 1:**
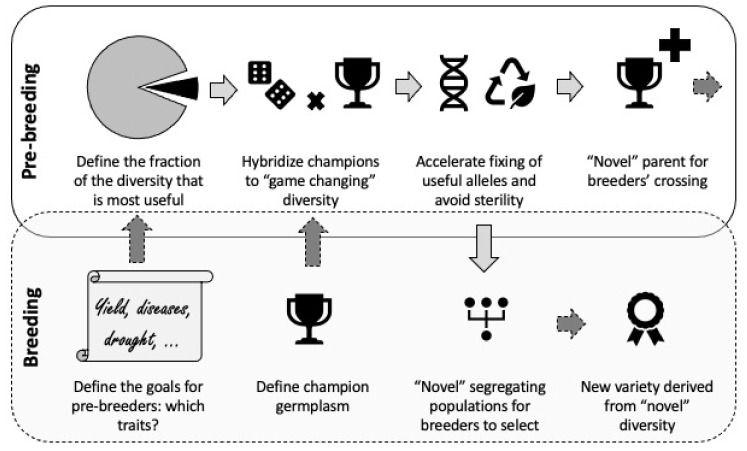
The key elements for a successful pre-breeding program are often simpler than one would expect. The key is to interact closely with breeders to provide the ready-to-use diversity that is truly useful for breeding efforts, and in return, receive the latest and best elite germplasm that can be used for introgressions.

Introgression of useful alleles from PGR into cultivars presents several challenges and requires pre-breeding strategies [32,52,260]. Although most breeding programs focus on elite × elite crosses, pre-breeding aims to exploit the hidden variation in PGR through elite × PGR crosses. Pre-breeding involves the identification of desirable traits or genes from PGR such as landraces and CWR that cannot be used directly in breeding programs, and the transfer of these traits into well-adapted cultivars to develop an intermediate set of materials that can be used by plant breeders in specific breeding programs to develop new cultivars with a broad genetic base (Figure 1) [44,218]. Depending on the crop, the complexity of the traits and PGR to be used in the crossing program, the time and resources breeders can invest in exploiting PGR, and the expertise required to handle difficult-to-use PGR, pre-breeding activities may be carried out by a single person in the main breeding program or by different people. In any case, pre-breeding should not be considered to be a separate pipeline. Pre-breeding using PGR provides new variation for crop improvement and should always be integrated and aligned with the main breeding programs irrespectively of who is handling it [52,53,54]. Systematic and targeted pre-breeding efforts involve a deep understanding of the genetics of the crop and the related taxa, prioritization of traits for improvement, trait discovery using high-throughput phenotyping and molecular tools, and the introgression of traits into a cultivated background with minimal linkage drag. Successful pre-breeding programs ensure a continuous supply of beneficial genetic variation for further use in breeding programs.

Recently, the Excellence in Breeding Platform (EiB; [261]) proposed a common stage-gate system for managing the breeding pipeline so that the right products are delivered on time and adopted. Following this stage-gate process for better management of private and public sector breeding programs (https://excellenceinbreeding.org/blog/applying-stage-gates-better-manage-public-breeding-programs accessed on 23 August 2021), we propose a similar process for the efficient management of wheat pre-breeding programs using CWR and exotic landraces, based on the following stages [53]:

Stage 1. Trait prioritization: A list of critical traits has to be defined in collaboration with breeders, genebank managers, farmers, and end-users. Emphasis should be given to those traits for which genetic diversity is lacking or limited in the cultivated germplasm or in the breeders’ working collections.

Stage 2. Validation of screening methods for trait characterization: The identification of “novel” traits often requires a step towards determining the best screening methods to identify them. This may involve the revision of already available methods to be applicable to diverse PGR on a larger scale, the establishment of precise phenotypic techniques in the field or under controlled conditions, or the use of molecular tools.

Stage 3. Screening of PGR for the trait of interest: Knowledge of the trait sought will help guide the identification of potentially promising PGR. This is usually done in a ranking order from the primary gene pool to the secondary to the tertiary gene pool, if possible, and in some extreme cases even to phylogenetically unrelated (*trans genesis*) species. However, it is critical to understand that Stage 2 and Stage 3 are closely connected, as phenotyping methods are often species-specific. Collaboration with genebank managers and the use of already available germplasm are of strategic importance. Heterogeneity is common within and between genebank accessions [262,263] and can disrupt genotype-phenotype connectivity when different samples from the same accession are used for different types of characterization. To avoid this, it is important to identify diverse, stable, and promising donors before initiating a crossing program. This can be achieved by precise characterization and evaluation of PGR under controlled environmental conditions and/or in target population environments using standardized protocols for two to three cycles following the single-seed descent method. The stable donors, referred to as trait-specific genetic stocks (TGS), can then be used in the crossing program. The use of small subsets such as core collections [264], mini core collections [265], reference sets [266], core reference sets [267], and the Focused Identification of Germplasm Strategy (FIGS) [268] have been established to facilitate the process of finding the diversity sought while screening a minimal number of PGR.

Stage 4. Germplasm enhancement: Often, outdated cytogenetic stocks have been used as recipient parents in pre-breeding. It is important to work with breeders to ensure that most promising “champion” germplasm representing the best and most recent elite breeding lines that are well-adapted to the target environment, is used as recipient parent in the crossing program (Figure 1). In this context, a positive trend has emerged for wheat in recent years. Old genotypes such as ‘Chinese Spring’ or ‘Opata’ for bread wheat or ‘Langdon’ for durum wheat were abandoned as parents for pre-breeding in favor of more modern cultivars. The use of biparental, multi-parental, bridge or other crossing schemes depends on the specificity of the PGR used as donors; this part is discussed in more detail in the following section. The outcome of this stage is segregating generations such as F_2_ or backcross progenies that can be used for further inbreeding and selection as well as for mapping studies.

Stage 5. Trait discovery: This is an optional stage where, depending upon the breeders’ objectives and availability of resources, segregating pre-breeding populations can be used to gain knowledge about the genetics of the trait(s), and used in genomics studies to determine marker-trait associations, etc.

Stage 6. Preliminary testing and multi-location evaluation: This stage depends on the requirements of variation in the main breeding program and requires close collaboration with the breeders. Pre-breeding material is included in the breeding pipeline from this stage onwards. It may involve the evaluation and selection process until stable lines are achieved that have been tested in the field for all major agronomic traits besides the trait of interest so that breeders can use them directly as parents. The final product will be promising introgression lines (ILs) that have the desired trait and minimum linkage drag. To identify the stable sources, the trait-specific ILs can be precisely evaluated in target environments. As mentioned above, close collaboration with breeders and farmers is required at this stage to select the best candidates.

Stage 7: Trait deployment: The process is completed at this stage and the “novel” traits in form of promising ILs, or entire pre-breeding populations are passed on to breeders. These stable sources are included in the breeding pipeline and promising ILs can also be conserved in genebanks for future use.

Systematic and targeted pre-breeding efforts will generate new diversity for use in wheat breeding programs. Molecular markers can be used to select difficult-to-measure traits and to increase the precision and efficiency of selection [269]. Various techniques are available for wheat, such as doubled-haploid production by anther or microspore culture, chromosome elimination using the wheat × maize system or the wheat × *Imperata cylindrica* system, and speed-breeding platforms for rapid generation turnover [270,271,272]. The use of these techniques will reduce the time required to develop a cultivar. Pre-breeding should become an integral part of crop breeding programs, including wheat, and should follow the proposed stage-gate approach to better manage pre-breeding pipelines.

## 7. Approaches to Facilitate Introgressions from PGR

**Figure 2:**
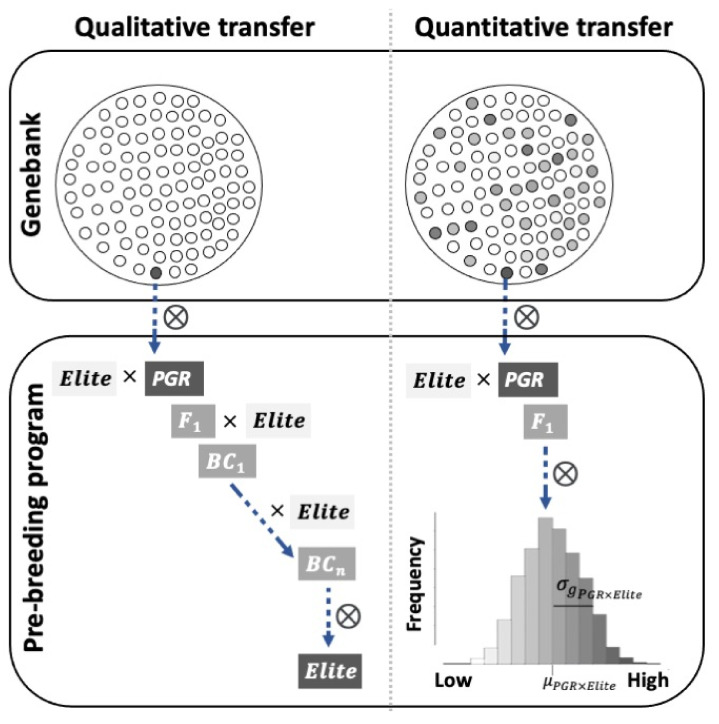
Strategies for introgressing variation from plant genetic resources (PGR) into (pre-)breeding programs to confer traits with simple (**left**) and polygenic (**right**) genetic architecture. For simple traits, backcrosses (BC) are commonly used for trait introgression. For polygenic traits, progeny selection within segregant inbred populations from F_1_ crosses is a common method for trait introgression. Each segregant inbred population is characterized by its mean ($\mu_{PGR\times Elite}$) and genetic standard deviation ($\sigma_{g_{PGR\times Elite}}$). The different shades of gray represent different values of trait variation at all levels of the scheme.

Several approaches have been proposed to facilitate the transfer of beneficial alleles from PGR. However, to determine the usefulness of these approaches, it is important to first understand the aim of the (pre-)breeder when using PGR. The selection of accessions and the genetic architecture of the targeted traits (i.e., simple or polygenic) determine which approach to use (Figure 2): (i) the introgression of a qualitative trait controlled by a single locus or a few loci from PGR; (ii) the introgression of a quantitative trait controlled by several loci from PGR; and (iii) the use of PGR to develop introgression lines for which any beneficial traits and their genetic basis will be determined later. In addition, there are several possible outputs. In some cases, the purpose of the introgression may be to understand the “modus operandi” of a gene. In most cases, the (pre-)breeder aims to produce materials for use as parental germplasm for further hybridizations, or in some cases even as direct cultivar release. The different approaches use different methods for genetic transfer, so they are described separately below.

### 7.1. Introgression of Qualitative Traits from PGR

This has been the case for many disease resistance alleles [273,274,275,276], but applies equally to any highly heritable trait largely under the influence of one, or a few, major gene loci. Classically, the trait is introduced by crossing and direct selection, leading to improvement of the elite germplasm. This process is effective but often lengthy because it involves several cycles of recurrent crossing and selection. More recently, molecular markers linked to traits have been used to enhance the precision and efficiency of the introgression process. Various genomic tools and other techniques, such as rapid generation turnover, single-seed descent and doubled-haploid methods allow the rapid development of elite germplasm enhanced by the introgression of carefully selected loci from the PGR (Figure 2, left) [269]. The non-molecular approach requires modest initial investment followed by years of steady progress through phenotypic selection, while the use of molecular markers requires up-front investment but then allows acceleration of screening. The deployment of transformation technologies such cis or trans genesis to introgress only the gene of interest holds great potential. However, due to the restrictions on commercialization of genetically modified cultivars in many countries, and high investment required, this approach is largely of academic interest only.

### 7.2. Introgression of Quantitative Traits from PGR

Quantitative traits usually exhibit strong genotype × environment (g×e) interaction, making phenotyping challenging and costly, while estimates for epistatic and non-additive interactions between alleles are usually biased. The use of genomic selection has often been presented as a strategic approach to facilitate introgression of quantitative traits [277]. Mathematical methods that efficiently deal with the “large p, small *n*” problem of modeling thousands-to-millions of molecular markers distributed throughout the genome in limited-sized populations were first proposed in the field of animal breeding [278,279]. These methods are known as genomic prediction, so the selection methods based on them are known as genomic selection. This methodology involves accurately phenotyping a sample of genotypes (the training population) and using this to calibrate a mathematical model made up of hundreds or thousands of genetic markers. This calibration process is key to the success of genomic selection, and involves repeated phenotyping, preferably across several environments and seasons, of a carefully chosen non-biased collection of ‘relevant’ genotypes. Each marker on the genotyping platform will be associated with a greater or lesser degree with the phenotype, and the model estimates a numerical effect for each of the markers. Thus, any genotype can be assigned a genomic-estimated breeding value (GEBV) by summing the estimates of all the markers used. In the right circumstances, this is a very valuable predictive tool and enables intensive selection at an early stage in the breeding process, therefore reducing the need for exhaustive field trials. However, useful predictions can only be made for alleles that are present in the training population. Unfortunately, almost by definition, the alleles of interest from PGR are unique and do not feature in existing models generated for modern lines. It is, therefore, necessary to run field trials for CWR and modern lines side by side. This presents a logistical challenge because of the differences in phenology and the required agronomic practices. Additionally, some risks need to be considered, including diseases and pests, weed escape, quarantine requirements, and wild traits such as shattering, dormancy, photoperiodism, and unfocused flowering. In some cases, it is difficult to access a genotyping platform that captures allelic diversity equally in PGR and elite cultivars [280,281].

Vanavermaete et al. [55] addressed these issues and proposed a multi-layer approach called “deep scoping” that keeps PGR and modern germplasm separate when modeling their relative contribution to GEBV, including specific factors to assess genetic diversity. A simpler approach would be to develop two training populations; one consisting of the PGR of interest together with other entries of the same species; and the other consisting only of modern germplasm. In any case, two separate models would have to be developed; one targeting the quantitative trait of interest in the PGR, and the other targeting the favorable alleles present in the modern germplasm used for the hybridization, as a way of performing foreground selection.

In principle, the ultimate question when attempting a PGR-introgression is: “Which cross (parent combination) will result in the best progenies?” In quantitative genetics, this question is essentially summarized by the “usefulness criterion” (UC) [282]. To explain this, the following scenario of polygenic trait introgression is assumed: the selected PGR (among many) shall be crossed with an elite line (Elite×PGR cross) and the superior inbred progenies will be identified (Figure 2, right). In this context, the UC can be defined as follows [282]:

$$UC_{Elite\times PGR}=\;\mu_{Elite\times PGR}+i\times h\times \sigma_{g_{Elite\times PGR}},$$

where $UC_{Elite\times PGR}$ is the UC of the particular Elite×PGR cross, $\mu_{Elite\times PGR}$ is the mean of an inbred population originated from the Elite×PGR cross, i is the selection intensity, h is the square root of the operational trait heritability according to testing capacities within a breeding program, and $\sigma_{g_{Elite\times PGR}}$ is the genetic standard deviation of inbred populations from the Elite×PGR cross. Inbred populations can be developed by recurrent selfing, or in a single generation using doubled-haploid technology [283] or the speed-breeding platform. Since (pre-)breeders can rarely increase selection intensity (*i*) without further investment [284], and the heritability (*h*) of a trait is in practice determined by its genotype × environment interaction and the trial error at the field station [285,286], only those crosses with either high $\mu_{Elite\times PGR}$, high $\sigma_{g_{Elite\times PGR}}$, or ideally both, are interesting for breeders. In the era of genomic prediction, $\mu_{Elite\times PGR}$ can be predicted as the average of the GEBV of the parents [287,288]. As a linear combination of predictions, the accuracy of $\mu_{Elite\times PGR}$ prediction will be directly affected by the accuracy of genomic prediction models, and by the reliability of the individual GEBV of parents [289]. Methods have been proposed to predict $\sigma_{g_{Elite\times PGR}}$ based on real parent genotypes, genomic-estimated additive effects of markers, and simulated progeny genotypes [290,291] Alternatively, progeny genotypes can be derived from the expected covariance between loci, which is a function of the expected recombination rates in different selfing generations and the linkage disequilibrium observed in the parents [287,288]. Potentially, but unexplored so far, the accuracy of $\sigma_{g_{Elite\times PGR}}$ prediction could be further improved by considering genomic models that handle epistatic interactions among markers during the estimation of effects [292,293,294]. Essentially, in the case of PGR-derived material, there is novelty in the genome that cannot be fully or even partially estimated from an understanding of the elite germplasm alone or from the native PGR accessions alone. We emphasize here that direct assessment of quantitative traits in a PGR compared to elite material can result in several ascertainment biases, and the same applies to genomic predictions. So far, we are not aware of any clear examples of successful transfer of quantitative traits from PGR by genomic selection, but it is theoretically possible.

### 7.3. Introgressions from PGR without Known Characteristics

As unfocused as this approach may sound, this method has some clear benefits and merits, as described below. As it is extremely difficult to predict elite × PGR crosses based on the available genetic value of the two parents, the use of PGR ”without known traits“ is widespread and successful. It is logistically and physiologically very challenging to perform effective field characterization of PGR for quantitative traits. Therefore, most of the information available for PGR is limited to qualitative traits. In addition, several authors have reported that genes of interest from PGR can be silenced or rendered ineffective after transfer to modern germplasm [295]. For example, Szabó-Hever et al. [205] showed that the resistance of synthetic bread wheat lines to *Fusarium* head blight could not always be predicted based on the response of the PGR used to produce the synthetics. Consistent with this, Merchuk-Ovnat et al. [296] transferred quantitative trait loci related to increased productivity under drought from wild emmer wheat to durum and bread wheat cultivars, and found that the expression of the different alleles was cultivar-specific. The most commonly used method is therefore to cross with PGR and then select based on performance, as it has been shown that breeding selection is ultimately the best approach for pyramiding quantitative traits. The use of top-crosses (A/PGR//B) or backcrosses (A/PGR//A) is considered best to reduce the total amount of PGR genome carried by the resulting progenies [49,287]. Singh et al. [45] applied this principle and confirmed through multi-location testing that many of the new introgressions matched the best performing elites. Another example is the release of the Moroccan variety ‘Nachit’ with 20% larger seed size than the original parents through a simple top-cross involving wild emmer and durum wheat genotypes [11,48].

## 8. Global Initiatives for Promoting the Use of PGR for Crop Improvement

Three of the most extensive examples of the introgression of PGR into modern wheat germplasm are: (i) the “Wheat Improvement Strategic Programme” (WISP, http://www.cerealsdb.uk.net/cerealgenomics/WISP/Consortium/WISP.php accessed on 21 September 2021) funded by the Biotechnology and Biological Sciences Research Council (BBRSC); (ii) the initiative “Adapting Agriculture to Climate Change: Collecting, Protecting and Preparing Crop Wild Relatives” (hereafter CWR Project), which is supported by the Government of Norway and managed by the Global Crop Diversity Trust (https://www.cwrdiversity.org/ accessed on 21 September 2021); and (iii) the “Seeds of Discovery” initiative for the production and use of landraces and synthetics in wheat managed by CIMMYT and funded by the government of Mexico (https://seedsofdiscovery.org accessed on 21 September 2021)).

A long-term commitment is needed to identify beneficial alleles of PGR and transfer them into breeding pipelines. A unique example of a long-term major pre-breeding initiative in various crops is the CWR Project, (https://www.cwrdiversity.org/project/pre-breeding/ accessed on 21 September 2021). Under this initiative, pre-breeding projects have been completed or are underway for 19 crops. The project involves more than 100 national and international partners including universities, non-governmental organizations, and seed clubs in 50 countries. The CWR Project has a strong emphasis on capacity building. Many of the pre-breeding partnerships are well advanced and will soon (after 2021) deliver promising CWR-derived pre-bred lines.

## 9. Summary and Conclusions

In the early 1960s, dwarfing genes introgressed from ‘Norin 10’ changed wheat cultivation worldwide. However, the deteriorating climatic conditions and other challenges have since put an end to the rapid genetic improvement. A new need has arisen to find the next breakthrough traits and the greatest hope is to find them in the vast PGR collections. A better understanding of the taxonomy and phylogenetic history of wheat paves the way for a better use of these collections. Here, we have presented several examples of successful incorporation of PGR for wheat improvement, and discussed promising introgression schemes. Given the heterogeneity within and between germplasm accessions stored in genebanks, it is important to develop TGS that can be used to generate new populations. These TGS are the immortal germplasm and are ideal for trait mapping, identification of candidate genes and re-sequencing studies, as well as for the development of a super-pangenome for wheat. We are also seeing a shift in the mindset of breeders, who are moving away from the “last resource” concept that was associated with the use of PGR and of CWR in the past. The establishment of pre-breeding pipelines has contributed to this change, and we have proposed here an integrated approach to align pre-breeding and breeding.

Broadening the genetic base of the elite wheat germplasm is then no longer just a means, but a real necessity to further increase genetic gain and to especially open new markets. Those breeding programs that succeed in integrating a strong pre-breeding pipeline are likely to be the real game changers in the years to come, able to develop varieties that are ready for the climatic challenges. Indeed, the success of these pre-breeding efforts depends on careful planning and efficient implementation, frequent monitoring to identify challenges at each step and measure results and impact, and strong networking and collaboration between the public and private sectors. Because of the costs and complexity associated with pre-breeding, now more than ever the role of public sector organizations and institutions becomes critical to provide freely novel alleles ready to be deployed. Donors have demonstrated good understanding of this need and have shown their willingness to drive this change.

## Figures and Tables

**Table 1:** Genebanks holding important *Triticum* and *Aegilops* collections, according to the historical FAO WIEWS database (http://www.fao.org/wiews-archive/wiews.jsp accessed on 23 August 2021), and present-day information from Genesys (https://www.genesys-pgr.org/ accessed on 23 August 2021) and EURISCO (https://eurisco.ipk-gatersleben.de accessed on 23 August 2021).

Country	Institute	Institute Acronym (FAO WIEWS Institure Code)	Number of Accessions	Information Source
***Triticum***				
Mexico	International Maize and Wheat Improvement Center	CIMMYT (MEX002)	142,484	Genesys
United States of America	National Small Grains Germplasm Research Facility, USDA-ARS	NSGC (USA029)	63,941	Genesys
Australia	Australian Grains Genebank, Department of Economic Development Jobs Transport and Resources	AGG (AUS165)	41,154	Genesys
China	Institute of Crop Science, Chinese Academy of Agricultural Sciences	ICS-CAAS (CHN001)	41,030	FAO WIEWS
Lebanon	International Centre for Agricultural Research in Dry Areas	ICARDA (LBN002)	38,897	Genesys
Russian Federation	N.I. Vavilov All-Russian Scientific Research Institute of Plant Genetic Resources	VIR (RUS001)	38,315	Genesys
India	National Bureau of Plant Genetic Resources	NBPGR (IND001)	35,889	FAO WIEWS
Japan	Department of Genetic Resources I, National Institute of Agrobiological Sciences	NIAS (JPN003)	34,652	FAO WIEWS
Italy	Istituto di Bioscienze e Biorisorse, Consiglio Nazionale delle Ricerche, Bari	IBBR-CNR (ITA436)	29,680	https://ibbr.cnr.it/mgd accessed on 23 August 2021
Germany	Genebank, Leibniz Institute of Plant Genetics and Crop Plant Research	IPK (DEU146)	27,442	Genesys, EURISCO
***Aegilops***				
Israel	Lieberman Germplasm Bank, Institute for Cereal Crops Improvement, Tel-Aviv University	ICCI-TELAVUN (ISR003)	7520	Genesys
Lebanon	International Centre for Agricultural Research in Dry Areas	ICARDA (LBN002)	5081	Genesys
Russian Federation	N.I. Vavilov All-Russian Scientific Research Institute of Plant Genetic Resources	VIR (RUS001)	3362	Genesys
Islamic Republic of Iran	National Plant Gene Bank of Iran, Seed and Plant Improvement Institute	NPGBI-SPII (IRN029)	2653	FAO WIEWS
Japan	Department of Genetic Resources I, National Institute of Agrobiological Sciences	NIAS (JPN003)	2433	FAO WIEWS
Japan	Plant Germplasm Institute, Faculty of Agriculture, Kyoto University	KYOPGI (JPN001)	2396	FAO WIEWS
United States of America	National Small Grains Germplasm Research Facility, USDA-ARS	NSGC (USA029)	2245	Genesys
Mexico	International Maize and Wheat Improvement Center	CIMMYT (MEX002)	2203	Genesys

## Data Availability

Not applicable.

## References

[1] IDRC *Facts and Figures on Food and Biodiversity*; Ottawa, Canada, 2010. https://www.idrc.ca/en/research-in-action/facts-figures-food-and-biodiversity.

[2] (2019). FAOSTAT. http://www.fao.org/faostat/en/#data/QC.

[3] European Union (2018). Production, Yields and Productivity.

[4] Feldman M., Bonjean A.P., Angus W.J. (2001). Origin of cultivated wheat. The World Wheat Book. A History of Wheat Breeding.

[5] Le Gouis J., Oury F.-X., Charmet G. (2020). How changes in climate and agricultural practices influenced wheat production in Western Europe. J. Cereal Sci..

[6] Tidiane Sall A., Chiari T., Legesse W., Seid-Ahmed K., Ortiz R., van Ginkel M., Bassi F.M. (2019). Durum wheat (*Triticum durum* Desf.): Origin, cultivation and potential expansion in Sub-Saharan Africa. Agronomy.

[7] Alsaleh A., Baloch F.S., Nachit M., Özkan H. (2016). Phenotypic and genotypic intra-diversity among Anatolian durum wheat “Kunduru” landraces. Biochem. Syst. Ecol..

[8] Khan H., Qureshi A.M.I., Dar Z.A., Wani S.H. (2019). Genetic improvement for end-use quality in wheat. Quality Breeding in Field Crops.

[9] Mastrangelo A.M., Cattivelli L. (2021). What makes bread and durum wheat different?. Trends Plant Sci..

[10] Mackay T.F.C., Falconer D.S. (1996). Introduction to Quantitative Genetics.

[11] Bassi F.M., Nachit M.M. (2019). Genetic gain for yield and allelic diversity over 35 years of durum wheat breeding at ICARDA. Crop Breed. Genet. Genom..

[12] Tadesse W., Sanchez-Garcia M., Assefa S.G., Amri A., Bishaw Z., Ogbonnaya F.C., Baum M. (2019). Genetic gains in wheat breeding and its role in feeding the world. Crop Breed. Genet. Genom..

[13] Austin R.B., Morgan C.L., Ford M.A., Blackwell R.D. (1980). Contributions to grain yield from pre-anthesis assimilation in tall and dwarf barley phenotypes in two contrasting seasons. Ann. Bot..

[14] Borlaug N.E. Wheat breeding and its impact on world food supply. Proceedings of the 3rd International Wheat Genetics Symposium.

[15] Laidig F., Piepho H.-P., Drobek T., Meyer U. (2014). Genetic and non-genetic long-term trends of 12 different crops in German official variety performance trials and on-farm yield trends. Theor. Appl. Genet..

[16] Slafer G., Kernich G. (1996). Have changes in yield (1900-1992) been accompanied by a decreased yield stability in Australian cereal production?. Austral. J. Agric. Res..

[17] Voss-Fels K.P., Stahl A., Wittkop B., Lichthardt C., Nagler S., Rose T., Chen T.-W., Zetzsche H., Seddig S., Majid Baig M. (2019). Breeding improves wheat productivity under contrasting agrochemical input levels. Nat. Plants.

[18] Grassini P., Eskridge K.M., Cassman K.G. (2013). Distinguishing between yield advances and yield plateaus in historical crop production trends. Nat. Commun..

[19] Juroszek P., von Tiedemann A. (2013). Climate change and potential future risks through wheat diseases: A review. Eur. J. Plant Pathol.

[20] Ray D.K., Ramankutty N., Mueller N.D., West P.C., Foley J.A. (2012). Recent patterns of crop yield growth and stagnation. Nat. Commun..

[21] Gerard G.S., Crespo-Herrera L.A., Crossa J., Mondal S., Velu G., Juliana P., Huerta-Espino J., Vargas M., Rhandawa M.S., Bhavani S. (2020). Grain yield genetic gains and changes in physiological related traits for CIMMYT’s High Rainfall Wheat Screening Nursery tested across international environments. Field Crops Res..

[22] Khoury C.K., Brush S., Costich D.E., Curry H., de Haan S., Engels J.M.M., Guarino L., Hoban S., Mercer K.L., Miller A.J. (2021). Crop genetic erosion: Understanding and responding to loss of crop diversity. New Phytol..

[23] Rutkoski J.E. (2019). Estimation of realized rates of genetic gain and indicators for breeding program assessment. Crop Sci..

[24] Cobb J.N., Juma R.U., Biswas P.S., Arbelaez J.D., Rutkoski J., Atlin G., Hagen T., Quinn M., Ng E.H. (2019). Enhancing the rate of genetic gain in public-sector plant breeding programs: Lessons from the breeder’s equation. Theor. Appl. Genet..

[25] Ru S., Bernardo R. (2019). Targeted recombination to increase genetic gain in self-pollinated species. Theor. Appl. Genet..

[26] Voss-Fels K.P., Cooper M., Hayes B.J. (2019). Accelerating crop genetic gains with genomic selection. Theor. Appl. Genet..

[27] van Ginkel M., Ortiz R. (2018). Cross the best with the best, and select the best: HELP in breeding selfing crops. Crop Sci..

[28] Girma E. (2017). Genetic erosion of wheat (*Triticum* spp.): Concept, research, results and challenges. J. Nat. Sci. Res..

[29] Asseng S., Ewert F., Martre P., Rötter R.P., Lobell D.B., Cammarano D., Kimball B.A., Ottman M.J., Wall G.W., White J.W. (2015). Rising temperatures reduce global wheat production. Nat. Clim. Chang..

[30] Daloz A.S., Rydsaa J.H., Hodnebrog Ø., Sillmann J., van Oort B., Mohr C.W., Agrawal M., Emberson L., Stordal F., Zhang T. (2021). Direct and indirect impacts of climate change on wheat yield in the Indo-Gangetic plain in India. J. Agric. Food Res..

[31] Miedaner T., Juroszek P. (2021). Climate change will influence disease resistance breeding in wheat in Northwestern Europe. Theor. Appl. Genet..

[32] Dempewolf H., Baute G., Anderson J., Kilian B., Smith C., Guarino L. (2017). Past and future use of wild relatives in crop breeding. Crop Sci..

[33] Hasan M., Hasibuzzaman A.S.M., Abdullah H.M., Kallol M.M.H., Salgotra R.K., Zargar S.M. (2020). Genetic and genomic resources and their exploitation for unlocking genetic potential from the wild relatives. Rediscovery of Genetic and Genomic Resources for Future Food Security.

[34] Nair K.P. (2019). Utilizing CWRs in major food crops to combat global warming. Combating Global Warming: The Role of Crop Wild Relatives for Food Security.

[35] Philipp N., Weichert H., Bohra U., Weschke W., Schulthess A.W., Weber H. (2018). Grain number and grain yield distribution along the spike remain stable despite breeding for high yield in winter wheat. PLoS ONE.

[36] Preece C., Peñuelas J. (2020). A return to the wild: Root exudates and food security. Trends Plant Sci..

[37] Prohens J., Gramazio P., Plazas M., Dempewolf H., Kilian B., Díez M.J., Fita A., Herraiz F.J., Rodríguez-Burruezo A., Soler S. (2017). Introgressiomics: A new approach for using crop wild relatives in breeding for adaptation to climate change. Euphytica.

[38] Sharma M., Punya, Gupta B.B., Salgotra R.K., Zargar S.M. (2020). Role of wild relatives for development of climate-resilient varieties. Rediscovery of Genetic and Genomic Resources for Future Food Security.

[39] Soriano J.M., Villegas D., Aranzana M.J., García del Moral L.F., Royo C. (2016). Genetic structure of modern durum wheat cultivars and Mediterranean landraces matches with their agronomic performance. PLoS ONE.

[40] Zaïm M., El Hassouni K., Gamba F., Filali-Maltouf A., Belkadi B., Sourour A., Amri A., Nachit M., Taghouti M., Bassi F.M. (2017). Wide crosses of durum wheat (*Triticum durum* Desf.) reveal good disease resistance, yield stability, and industrial quality across Mediterranean sites. Field Crops Res..

[41] Schmolke M., Zimmermann G., Buerstmayr H., Schweizer G., Miedaner T., Korzun V., Ebmeyer E., Hartl L. (2005). Molecular mapping of *Fusarium* head blight resistance in the winter wheat population Dream/Lynx. Theor. Appl. Genet..

[42] Schmolke M., Zimmermann G., Schweizer G., Miedaner T., Korzun V., Ebmeyer E., Hartl L. (2008). Molecular mapping of quantitative trait loci for field resistance to *Fusarium* head blight in a European winter wheat population. Plant Breed..

[43] Zhu Z., Hao Y., Mergoum M., Bai G., Humphreys G., Cloutier S., Xia X., He Z. (2019). Breeding wheat for resistance to *Fusarium* head blight in the Global North: China, USA, and Canada. Crop J..

[44] Kilian B., Dempewolf H., Guarino L., Werner P., Coyne C., Warburton M.L. (2021). Crop Science special issue: Adapting agriculture to climate change: A walk on the wild side. Crop Sci..

[45] Singh S., Vikram P., Sehgal D., Burgueño J., Sharma A., Singh S.K., Sansaloni C.P., Joynson R., Brabbs T., Ortiz C. (2018). Harnessing genetic potential of wheat germplasm banks through impact-oriented-prebreeding for future food and nutritional security. Sci. Rep..

[46] Leišová-Svobodová L., Michel S., Tamm I., Chourová M., Janovska D., Grausgruber H. (2019). Diversity and pre-breeding prospects for local adaptation in oat genetic resources. Sustainability.

[47] Rubio Teso M.L., Iriondo J.M. (2019). *In situ* conservation assessment of forage and fodder CWR in Spain using phytosociological associations. Sustainability.

[48] El Haddad N., Kabbaj H., Zaïm M., El Hassouni K., Tidiane Sall A., Azouz M., Ortiz R., Baum M., Amri A., Gamba F. (2021). Crop wild relatives in durum wheat breeding: Drift or thrift?. Crop Sci..

[49] Sharma S., Paul P.J., Kumar C.V.S., Rao P.J., Prashanti L., Muniswamy S., Sharma M. (2019). Evaluation and identification of promising introgression lines derived from wild *Cajanus* species for broadening the genetic base of cultivated pigeonpea [*Cajanus cajan* (L.) Millsp.]. Front. Plant Sci..

[50] Aberkane H., Kishii M., Amri A., Payne T.S., Smale M., Jamora N. (2019). Reaching into the Past to Tackle New Challenges: Improving Wheat by Conserving Wild ‘Goat Grass’.

[51] Hoisington D., Khairallah M., Reeves T., Ribaut J.V., Skovmand B., Taba S., Warburton M. (1999). Plant genetic resources: What can they contribute toward increased crop productivity?. Proc. Natl. Acad. Sci. USA.

[52] Sharma S. (2017). Prebreeding using wild species for genetic enhancement of grain legumes at ICRISAT. Crop Sci..

[53] Sharma S., Sharma R., Govindaraj M., Mahala R.S., Satyavathi C.T., Srivastava R.K., Gumma M.K., Kilian B. (2021). Harnessing wild relatives of pearl millet for germplasm enhancement: Challenges and opportunities. Crop Sci..

[54] Sharma S., Sharma R., Pujar M., Yadav D., Yadav Y., Rathore A., Mahala R.S., Singh I., Verma Y., Deora V.S. (2021). Use of wild *Pennisetum* species for improving biotic and abiotic stress tolerance in pearl millet. Crop Sci..

[55] Vanavermaete D., Fostier J., Maenhout S., De Baets B. (2021). Deep scoping: A breeding strategy to preserve, reintroduce and exploit genetic variation. Theor. Appl. Genet..

[56] FAO. www.fao.org/wiews-archive/germplasm_report.jsp.

[57] Knüpffer H., Muehlbauer G.J., Feuillet C. (2009). Triticeae genetic resources in ex situ genebank collections. Genetics and Genomics of the Triticeae.

[58] Kimber G., Feldman M. (1987). Wild Wheat, an Introduction.

[59] Kishii M. (2019). An update of recent use of *Aegilops* species in wheat breeding. Front. Plant Sci..

[60] Kilian B., Mammen K., Millet E., Sharma R., Graner A., Salamini F., Hammer K., Özkan H., Kole C. (2011). Aegilops. Wild Crop Relatives: Genomics and Breeding Resources. Cereals.

[61] Castaneda-Alvarez N.P., Khoury C.K., Achicanoy H.A., Bernau V., Dempewolf H., Eastwood R.J., Guarino L., Harker R.H., Jarvis A., Maxted N. (2016). Global conservation priorities for crop wild relatives. Nat. Plants.

[62] Shaw P.D., Weise S., Obreza M., Raubach S., Mccouch S., Kilian B., Werner P., Ghamkhar K., Williams W., Brown A.H.D. (2021). Database solutions for genebanks and germplasm collections. Plant Genetic Resources in the 21th Century. The OMICS Era.

[63] Weise S., Oppermann M., Maggioni L., van Hintum T., Knüpffer H. (2017). EURISCO: The European search catalogue for plant genetic resources. Nucleic Acids Res..

[64] Raubach S., Kilian B., Dreher K., Amri A., Bassi F.M., Boukar O., Cook D., Cruickshank A., Fatokun C., El Haddad N. (2021). From bits to bites: Advancement of the Germinate platform to support prebreeding informatics for crop wild relatives. Crop Sci..

[65] Shaw P.D., Raubach S., Hearne S.J., Dreher K., Bryan G., McKenzie G., Milne I., Stephen G., Marshall D.F. (2017). Germinate 3: Development of a common platform to support the distribution of experimental data on crop wild relatives. Crop Sci..

[66] Dash S., Campbell J.D., Cannon E.K.S., Cleary A.M., Huang W., Kalberer S.R., Karingula V., Rice A.G., Singh J., Umale P.E. (2016). Legume information system (LegumeInfo.org): A key component of a set of federated data resources for the legume family. Nucleic Acids Res..

[67] König P., Beier S., Basterrechea M., Schüler D., Arend D., Mascher M., Stein N., Scholz U., Lange M. (2020). BRIDGE—A visual analytics Web tool for barley genebank genomics. Front. Plant Sci..

[68] Dorofeev V.F., Filatenko A.A., Migushova E.F., Udachin R.A., Jakubziner M.M., Brezhnev D.D. (1979). Flora of Cultivated Plants, Volume 1, Wheat.

[69] Hammer K., Gladis T. (2014). Notes on infraspecific nomenclature and classifications of cultivated plants in Compositae, Cruciferae, Cucurbitaceae, Gramineae (with a remark on *Triticum dicoccon* Schrank) and Leguminosae. Genet. Resour. Crop Evol..

[70] Key J.M. (1988). A plant breeder’s perspective on taxonomy of cultivated plants. Biol. Zentralbl..

[71] Schiemann E. (1948). Weizen Roggen Gerste. Systematik, Geschichte und Verwendung.

[72] Van Slageren M.W. (1994). Wild Wheats: A Monograph of Aegilops L. and Amblyopyrum (Jaub. et Spach) Eig (Poaceae).

[73] Bowden W.M. (1959). The taxonomy and nomenclature of the wheats, barleys and ryes and their relatives. Can. J. Bot..

[74] Kimber G., Sears E.R. Assignment of genome symbols in the *Triticeae*. Proceedings of the 6th International Wheat Genetics Symposium.

[75] Chennaveeraiah M.S. (1960). Karyomorphologic and cytotaxonomic studies in *Aegilops*. Acta Horti Gotobg..

[76] Eig A. (1929). Monographisch-kritische Übersicht der Gattung *Aegilops*. Repert. Spec. Nov. Regni Veg. Beih. Berl..

[77] Hammer K. (1980). Vorarbeiten zur monographischen Darstellung von Wildpflanzensortimenten: *Aegilops* L.. Kulturpflanze.

[78] Hammer K. (1980). Zur Taxonomie und Nomenklatur der Gattung *Aegilops* L.. Feddes Rep..

[79] Kihara H. (1954). Considerations on the evolution and distribution of *Aegilops* species based on the analyser-method. Cytologia.

[80] Witcombe J.R. (1983). A Guide to the Species of Aegilops L. Their Taxonomy, Morphology and Distribution.

[81] Zhukovsky P.M. (1928). A critical-systematical survey of the species of the genus *Aegilops* L.. Bull. Appl. Bot. Genet. Plant Breed.

[82] Dvořák J. Genome analysis in the *Triticum-Aegilops* alliance. Proceedings of the 9th International Wheat Genetics Symposium.

[83] Kimber G., Tsunewaki K. Genome symbols and plasma types in the wheat group. Proceedings of the 7th International Wheat Genetics Symposium.

[84] Edet O.U., Gorafi Y.S.A., Nasuda S., Tsujimoto H. (2018). DArTseq-based analysis of genomic relationships among species of tribe *Triticeae*. Sci. Rep..

[85] Zeibig F., Kilian B., Frei M. (2021). The grain quality of wheat wild relatives in the evolutionary context. Theor. Appl. Genet..

[86] Bernhardt N., Brassac J., Dong X., Willing E.-M., Poskar C.H., Kilian B., Blattner F.R. (2020). Genome-wide sequence information reveals recurrent hybridization among diploid wheat wild relatives. Plant J..

[87] Bernhardt N., Brassac J., Kilian B., Blattner F.R. (2017). Dated tribe-wide whole chloroplast genome phylogeny indicates recurrent hybridizations within Triticeae. BMC Evol. Biol..

[88] Glémin S., Scornavacca C., Dainat J., Burgarella C., Viader V., Ardisson M., Sarah G., Santoni S., David J., Ranwez V. (2019). Pervasive hybridizations in the history of wheat relatives. Sci. Adv..

[89] Li L.-F., Zhang Z.-B., Wang Z.-H., Li N., Sha Y., Wang X.-F., Ding N., Li Y., Zhao J., Wu Y. (2021). Genome sequences of the five *Sitopsis* species of *Aegilops* and the origin of polyploid wheat B-subgenome. bioRxiv.

[90] Marcussen T., Sandve S.R., Heier L., Spannagl M., Pfeifer M., Jakobsen K.S., Wulff B.B.H., Steuernagel B., Mayer K.F.X., Olsen O.-A. (2014). Ancient hybridizations among the ancestral genomes of bread wheat. Science.

[91] Walkowiak S., Gao L., Monat C., Haberer G., Kassa M.T., Brinton J., Ramirez-Gonzalez R.H., Kolodziej M.C., Delorean E., Thambugala D. (2020). Multiple wheat genomes reveal global variation in modern breeding. Nature.

[92] Feldman M., Levy A.A., Molnár-Láng M., Ceoloni C., Doležel J. (2015). Origin and evolution of wheat and related Triticeae species. Alien Introgression in Wheat: Cytogenetics, Molecular Biology, and Genomics.

[93] Lilienfeld F.A. (1951). H. Kihara: Genome-analysis in *Triticum* and *Aegilops*. X. Concluding review. Cytologia.

[94] Tsunewaki K. (2009). Plasmon analysis in the *Triticum-Aegilops* complex. Breed. Sci..

[95] Huang S., Sirikhachornkit A., Su X., Faris J., Gill B., Haselkorn R., Gornicki P. (2002). Genes encoding plastid acetyl-CoA carboxylase and 3-phosphoglycerate kinase of the *Triticum/Aegilops* complex and the evolutionary history of polyploid wheat. Proc. Natl. Acad. Sci. USA.

[96] Middleton C.P., Senerchia N., Stein N., Akhunov E.D., Keller B., Wicker T., Kilian B. (2014). Sequencing of chloroplast genomes from wheat, barley, rye and their relatives provides a detailed insight into the evolution of the Triticeae tribe. PLoS ONE.

[97] Avni R., Lux T., Minz-Dub A., Millet E., Sela H., Distelfeld A., Deek J., Yu G., Steuernagel B., Pozniak C. (2021). Genome sequences of *Aegilops* species of section *Sitopsis* reveal phylogenetic relationships and provide resources for wheat improvement. bioRxiv.

[98] Dvořák J., Zhang H. (1990). Variation in repeated nucleotide sequences sheds light on the phylogeny of the wheat B and G genomes. Proc. Natl. Acad. Sci. USA.

[99] Ogihara Y., Tsunewaki K. (1988). Diversity and evolution of chloroplast DNA in *Triticum* and *Aegilops* as revealed by restriction fragment analysis. Theor. Appl. Genet..

[100] Sarkar P., Stebbins G.L. (1956). Morphological evidence concerning the origin of the B genome in wheat. Amer. J. Bot..

[101] Gornicki P., Zhu H., Wang J., Challa G.S., Zhang Z., Gill B.S., Li W. (2014). The chloroplast view of the evolution of polyploid wheat. New Phytol..

[102] Dvořák J., Akhunov E.D. (2005). Tempos of gene locus deletions and duplications and their relationship to recombination rate during diploid and polyploid evolution in the *Aegilops-Triticum* alliance. Genetics.

[103] Luo M.C., Yang Z.L., You F.M., Kawahara T., Waines J.G., Dvořák J. (2007). The structure of wild and domesticated emmer wheat populations, gene flow between them, and the site of emmer domestication. Theor. Appl. Genet..

[104] Mori N., Ishii T., Ishido T., Hirosawa S., Watatani H., Kawahara, Nesbitt M., Belay G., Takumi S., Ogihara Y. Origin of domesticated emmer and common wheat inferred from chloroplast DNA fingerprinting. Proceedings of the Proceedings of the 10th International Wheat Genetic Symposium.

[105] Özkan H., Brandolini A., Pozzi C., Effgen S., Wunder J., Salamini F. (2005). A reconsideration of the domestication geography of tetraploid wheats. Theor. Appl. Genet..

[106] Özkan H., Willcox G., Graner A., Salamini F., Kilian B. (2011). Geographic distribution and domestication of wild emmer wheat (*Triticum dicoccoides*). Genet. Resour. Crop Evol..

[107] Civáň P., Ivaničová Z., Brown T.A. (2013). Reticulated origin of domesticated emmer wheat supports a dynamic model for the emergence of agriculture in the Fertile Crescent. PLoS ONE.

[108] Maccaferri M., Harris N.S., Twardziok S.O., Pasam R.K., Gundlach H., Spannagl M., Ormanbekova D., Lux T., Prade V.M., Milner S.G. (2019). Durum wheat genome highlights past domestication signatures and future improvement targets. Nat. Genet..

[109] Pont C., Leroy T., Seidel M., Tondelli A., Duchemin W., Armisen D., Lang D., Bustos-Korts D., Goué N., Balfourier F. (2019). Tracing the ancestry of modern bread wheats. Nat. Genet..

[110] Gioia T., Nagel K.A., Beleggia R., Fragasso M., Ficco D.B., Pieruschka R., De Vita P., Fiorani F., Papa R. (2015). Impact of domestication on the phenotypic architecture of durum wheat under contrasting nitrogen fertilization. J. Exp. Bot..

[111] Vavilov N.I. (1951). The origin, variation, immunity and breeding of cultivated plants. Soil Sci..

[112] Kabbaj H., Sall A.T., Al-Abdallat A., Geleta M., Amri A., Filali-Maltouf A., Belkadi B., Ortiz R., Bassi F.M. (2017). Genetic diversity within a global panel of durum wheat (*Triticum durum*) landraces and modern germplasm reveals the history of alleles exchange. Front. Plant Sci..

[113] Martínez-Moreno F., Solís I., Noguero D., Blanco A., Özberk İ., Nsarellah N., Elias E., Mylonas I., Soriano J.M. (2020). Durum wheat in the Mediterranean Rim: Historical evolution and genetic resources. Genet. Resour. Crop Evol..

[114] Mazzucotelli E., Sciara G., Mastrangelo A.M., Desiderio F., Xu S.S., Faris J., Hayden M.J., Tricker P.J., Özkan H., Echenique V. (2020). The Global Durum Wheat Panel (GDP): An international platform to identify and exchange beneficial alleles. Front. Plant Sci..

[115] Mengistu D.K., Kidane Y.G., Catellani M., Frascaroli E., Fadda C., Pè M.E., Dell’Acqua M. (2016). High-density molecular characterization and association mapping in Ethiopian durum wheat landraces reveals high diversity and potential for wheat breeding. Plant Biotech. J..

[116] Mengistu D.K., Kiros A.Y., Pè M.E. (2015). Phenotypic diversity in Ethiopian durum wheat (*Triticum turgidum* var. durum) landraces. Crop J..

[117] Badaeva E.D., Badaev N.S., Gill B.S., Filatenko A.A. (1994). Intraspecific karyotype divergence in *Triticum araraticum* (Poaceae). Plant Syst. Evol..

[118] Nave M., Taş M., Raupp J., Tiwari V.K., Özkan H., Poland J., Hale I., Komatsuda T., Distelfeld A. (2021). The independent domestication of Timopheev’s wheat: Insights from haplotype analysis of the Brittle rachis 1 (*BTR1-A*) gene. Genes.

[119] Badaeva E.D., Konovalov F.A., Knüpffer H., Fricano A., Ruban A.S., Kehel Z., Zoshchuk S.A., Surzhikov S.A., Neumann K., Graner A. (2021). Genetic diversity, distribution and domestication history of the neglected GGA^t^A^t^ genepool of wheat. Theor. Appl. Genet..

[120] Kihara H. (1944). Die Entdeckung des DD-Analysators beim Weizen. Agric. Hortic. Tokio.

[121] McFadden E.S., Sears E.R. (1946). The origin of *Triticum spelta* and its free-threshing hexaploid relatives. J. Hered..

[122] Singh N., Wu S., Tiwari V., Sehgal S., Raupp J., Wilson D., Abbasov M., Gill B., Poland J. (2019). Genomic analysis confirms population structure and identifies inter-lineage hybrids in *Aegilops tauschii*. Front. Plant Sci..

[123] Dvořák J., Luo M.C., Yang Z.L., Zhang H.B. (1998). The structure of the *Aegilops tauschii* genepool and the evolution of hexaploid wheat. Theor. Appl. Genet..

[124] Wang J., Luo M.-C., Chen Z., You F.M., Wei Y., Zheng Y., Dvořák J. (2013). *Aegilops tauschii* single nucleotide polymorphisms shed light on the origins of wheat D-genome genetic diversity and pinpoint the geographic origin of hexaploid wheat. New Phytol..

[125] Badaeva E.D., Fisenko A.V., Surzhikov S.A., Yankovskaya A.A., Chikida N.N., Zoshchuk S.A., Belousova M.K., Dragovich A.Y. (2019). Genetic heterogeneity of a diploid grass *Aegilops tauschii* revealed by chromosome banding methods and electrophoretic analysis of the seed storage proteins (gliadins). Russ. J. Genet..

[126] Zhao L., Ning S., Yi Y., Zhang L., Yuan Z., Wang J., Zheng Y., Hao M., Liu D. (2018). Fluorescence *in situ* hybridization karyotyping reveals the presence of two distinct genomes in the taxon *Aegilops tauschii*. BMC Genom..

[127] Petersen G., Seberg O., Yde M., Berthelsen K. (2006). Phylogenetic relationships of *Triticum* and *Aegilops* and evidence for the origin of the A, B, and D genomes of common wheat (*Triticum aestivum*). Mol. Phyl. Evol..

[128] Renfrew J.M. (1973). Palaeoethnobotany—The Prehistoric Food Plants of the Near East and Europe.

[129] Badaeva E.D., Ruban A.S., Zoshchuk S.A., Surzhikov S.A., Knüpffer H., Kilian B. (2016). Molecular cytogenetic characterization of *Triticum timopheevii* chromosomes provides new insight on genome evolution of *T. zhukovskyi*. Plant Syst. Evol..

[130] Menabde V.L., Ericzjan A.A. (1960). To the investigation of Georgian wheat Zanduri. Proc. Georg. Acad. Sci..

[131] Mitrofanova O., Badaeva E., Salina E., Bonjean A., Angus W., van Ginkel J.M. (2016). *Triticum timopheevii*, *T. araraticum* and *T. zhukovskyi*, bread and durum wheat relatives carrying the G genome. The World Wheat Book: A History of Wheat Breeding.

[132] Genesys. https://www.genesys-pgr.org/a/v2R2VmqW782.

[133] Kilian B., Özkan H., Pozzi C., Salamini F., Muehlbauer G.J., Feuillet C. (2009). Domestication of the Triticeae in the Fertile Crescent. Genetics and Genomics of the Triticeae.

[134] Peng J.H., Sun D., Nevo E. (2011). Domestication evolution, genetics and genomics in wheat. Mol. Biol. Evol..

[135] Shewry P.R. (2009). Wheat. J. Exp. Bot..

[136] Zohary D., Hopf M., Weiss E. (2012). Domestication of plants in the Old World.

[137] Harlan J.R., de Wet J.M.J. (1971). Toward a rational classification of cultivated plants. Taxon.

[138] Feldman M., Sears E.R. (1981). The wild gene resources of wheat. Sci. Am..

[139] Jauhar P.P., Damania A.B. (1993). Alien gene transfer and genetic enrichment of wheat. Biodiversity and Wheat Improvement.

[140] Kihara H. (1924). Cytologische und genetische Studien bei wichtigen Getreidearten mit besonderer Rücksicht auf das Verhalten der Chromosomen und die Sterilität in den Bastarden. Mem. Cell. Sci. Kyoto Imp. Univ. Ser. B.

[141] Molnár-Láng M., Molnár-Láng M., Ceoloni C., Doležel J. (2015). The crossability of wheat with rye and other related species. Alien Introgression in Wheat.

[142] Molnár-Láng M., Molnár I., Szakács É., Linc G., Bedö Z., Tuberosa R., Graner A., Frison E. (2014). Production and molecular cytogenetic identification of wheat-alien hybrids and introgression lines. Genomics of Plant Genetic Resources. Volume 1. Managing, Sequencing and Mining Genetic Resources.

[143] Moore G. (2014). The control of recombination in wheat by *Ph1* and its use in breeding. Methods Mol. Biol..

[144] Mujeeb-Kazi A., Gilchrist L.I., Fuentes-Davila G., Delgado R., Jaradat A.A. (1997). Production and utilization of D-genome synthetic hexaploids in wheat improvement. Proceedings of the 3rd International Triticeae Symposium, ICARDA, Aleppo, Syria, 4-8 May 1997.

[145] Riley R., Unrau J., Chapman V. (1958). Evidence on the origin of the B genome of wheat. J. Hered..

[146] Sharma H.C., Gill B.S. (1983). Current status of wide hybridization in wheat. Euphytica.

[147] Salvi S., Porfiri O., Ceccarelli S. (2012). Nazareno Strampelli, the ‘Prophet’ of the green revolution. J. Agric. Sci..

[148] Borojevic K., Borojevic K. (2005). Historic role of the wheat variety Akakomugi in southern and central European wheat breeding programs. Breed. Sci..

[149] Dreisigacker S., Zhang P., Warburton M.L., Skovmand B., Hoisington D., Melchinger A.E. (2005). Genetic diversity among and within CIMMYT wheat landrace accessions investigated with SSRs and implications for plant genetic resources management. Crop Sci..

[150] Matsumoto T. (1968). Norin 10—A dwarf winter wheat variety. Jap. Agric. Res. Quart..

[151] Reitz L.P., Salmon S.C. (1968). Origin, history, and use of Norin 10 wheat. Crop Sci..

[152] Lopes M.S., El-Basyoni I., Baenziger P.S., Singh S., Royo C., Ozbek K., Aktas H., Ozer E., Ozdemir F., Manickavelu A. (2015). Exploiting genetic diversity from landraces in wheat breeding for adaptation to climate change. J. Exp. Bot..

[153] Hede A.R., Skovmand B., Reynolds M.P., Crossa J., Vilhelmsen A.L., Stolen O. (1999). Evaluating genetic diversity for heat tolerance traits in Mexican wheat landraces. Genet. Resour. Crop Evol..

[154] Vikram P., Franco J., Burgueno-Ferreira J., Li H.H., Sehgal D., Saint Pierre C., Ortiz C., Sneller C., Tattaris M., Guzman C. (2016). Unlocking the genetic diversity of Creole wheats. Sci. Rep..

[155] Royo C., Briceño-Félix G.A., Bonjean A.P., Angus W.J., van Ginkel M. (2011). Spanish wheat pool. The World Wheat Book: A History of Wheat Breeding.

[156] Taranto F., D’Agostino N., Rodriguez M., Pavan S., Minervini A.P., Pecchioni N., Papa R., De Vita P. (2020). Whole genome scan reveals molecular signatures of divergence and selection related to important traits in durum wheat germplasm. Front. Genet..

[157] Bieńkowska T., Suchowilska E., Wiwart M. (2020). *Triticum polonicum* L. as promising source material for breeding new wheat cultivars. J. Elem..

[158] Bienkowska T., Suchowilska E., Kandler W., Krska R., Wiwart M. (2019). *Triticum polonicum* L. as potential source material for the biofortification of wheat with essential micronutrients. Plant Genet. Resour.-Charact. Util..

[159] Chen Q., Kang H.-Y., Fan X., Wang Y., Sha L.-N., Zhang H.-Q., Zhong M.-Y., Xu L.-L., Zeng J., Yang R.-W. (2013). Evolutionary history of *Triticum petropavlovskyi* Udacz. et Migusch. inferred from the sequences of the 3-phosphoglycerate kinase gene. PLoS ONE.

[160] Kang H.Y., Fan X., Zhang H.Q., Sha L.N., Sun G.L., Zhou Y.H. (2010). The origin of *Triticum petropavlovskyi* Udacz. et Migusch.: Demonstration of the utility of the genes encoding plastid acetyl-CoA carboxylase sequence. Mol. Biol. Evol..

[161] Akond A.S.M.G.M., Watanabe N. (2005). Genetic variation among Portuguese landraces of ‘Arrancada’ wheat and *Triticum petropavlovskyi* by AFLP-based assessment. Genet. Resour. Crop Evol..

[162] Martynov S.P., Dobrotvorskaya T.V., Krupnov V.A. (2018). Analysis of the distribution of *Triticum timopheevii* Zhuk. genetic material in common wheat varieties (*Triticum aestivum* L.). Russ. J. Genet..

[163] McIntosh R.A., Yamazaki Y., Dubkovsky G., Rogers J., Morris C.F., Appels R., Xia X.C. Catalogue of gene symbols for wheat. Proceedings of the 12th International Wheat Genetics Symposium.

[164] Maan S.S., Lucken K.A. (1972). Interacting male sterility-male fertility restoration systems for hybrid wheat research. Crop Sci..

[165] Mikó P., Megyeri M., Kovács G. Characterization of Triticum timopheevii Zhuk. gene bank accessions to gain useful materials for organic wheat breeding. Proceedings of the Agrisafe Final Conference, Climate Change: Challenges and Opportunities in Agriculture.

[166] Wurschum T., Leiser W.L., Weissmann S., Maurer H.P. (2017). Genetic architecture of male fertility restoration of *Triticum timopheevii* cytoplasm and fine-mapping of the major restorer locus *Rf3* on chromosome 1B. Theor. Appl. Genet..

[167] Villareal R.L., Rajaram S., Mujeeb-Kazi A., Del Toro E. (1991). The effect of chromosome 1B/1R translocation on the yield potential of certain spring wheats (*Triticum aestivum* L.). Plant Breed..

[168] Schlegel G., Schlegel R. (1989). A compendium of reciprocal intervarietal translocations in hexaploid wheat. Genet. Resour. Crop Evol..

[169] Friebe B., Jiang J., Raupp W.J., McIntosh R.A., Gill B.S. (1996). Characterization of wheat-alien translocations conferring resistance to diseases and pests: Current status. Euphytica.

[170] Rabinovich S.V. (1998). Importance of wheat-rye translocations for breeding modern cultivar of *Triticum aestivum* L.. Euphytica.

[171] Badaeva E.D., Dedkova O.S., Gay G., Pukhalskyi V.A., Zelenin A.V., Bernard S., Bernard M. (2007). Chromosomal rearrangements in wheat: Their types and distribution. Genome.

[172] Ren T.H., Chen F., Yan B.J., Zhang H.Q., Ren Z.L. (2010). Genetic diversity of wheat-rye 1BL.1RS translocation lines derived from different wheat and rye sources. Euphytica.

[173] Skovmand B., Villareal R.L., van Ginkel M., Rajaram S., Ferrara G.O. (1997). Semidwarf Bread Wheats: Names, Parentages, Pedigrees and Origins.

[174] Doussinault G., Delibes A., Sanchez-Monge R., Garcia-Olmedo F. (1983). Transfer of a dominant gene for resistance to eyespot disease from a wild grass to hexaploid wheat. Nature.

[175] Bariana H.S., McIntosh R.A. (1994). Characterisation and origin of rust and powdery mildew resistance genes in VPM1 wheat. Euphytica.

[176] Cruz C.D., Peterson G.L., Bockus W.W., Kankanala P., Dubcovsky J., Jordan K.W., Akhunov E., Chumley F., Baldelomar F.D., Valent B. (2016). The 2NS translocation from *Aegilops ventricosa* confers resistance to the *Triticum* pathotype of *Magnaporthe oryzae*. Crop Sci..

[177] Cruz C.D., Valent B. (2017). Wheat blast disease: Danger on the move. Trop. Plant Pathol..

[178] Gao L., Koo D.-H., Juliana P., Rife T., Singh D., Lemes da Silva C., Lux T., Dorn K.M., Clinesmith M., Silva P. (2021). The *Aegilops ventricosa* 2N^v^S segment in bread wheat: Cytology, genomics and breeding. Theor. Appl. Genet..

[179] Singh D., Wang X., Kumar U., Gao L., Noor M., Imtiaz M., Singh R.P., Poland J. (2019). High-throughput phenotyping enabled genetic dissection of crop lodging in wheat. Front. Plant Sci..

[180] Schneider A., Molnár I., Molnár-Láng M. (2008). Utilisation of *Aegilops* (goatgrass) species to widen the genetic diversity of cultivated wheat. Euphytica.

[181] Olivera P.D., Rouse M.N., Jin Y. (2018). Identification of new sources of resistance to wheat stem rust in *Aegilops* spp. in the tertiary genepool of wheat. Front. Plant Sci..

[182] Gill B.S., Sharma C., Raupp W.J., Browder L.E., Heachett J.H., Harvey T.L., Moseman J.G., Waines J.G. (1985). Evaluation of *Aegilops* species for resistance to wheat powdery mildew, wheat leaf rust, Hessian fly, and greenbug. Plant Dis..

[183] Rawat N., Schoen A., Singh L., Mahlandt A., Wilson D.L., Liu S., Lin G., Gill B.S., Tiwari V.K. (2018). TILL-D: An *Aegilops tauschii* TILLING resource for wheat improvement. Front. Plant Sci..

[184] Suneja Y., Gupta A.K., Bains N.S. (2019). Stress adaptive plasticity: *Aegilops tauschii* and *Triticum dicoccoides* as potential donors of drought associated morpho-physiological traits in wheat. Front. Plant Sci..

[185] Feuillet C., Langridge P., Waugh R. (2008). Cereal breeding takes a walk on the wild side. Trends Genet..

[186] Martín A.C., Rey M.D., Shaw P., Moore G. (2017). Dual effect of the wheat *Ph1* locus on chromosome synapsis and crossover. Chromosoma.

[187] Rey M.D., Martín A.C., Higgins J., Swarbreck D., Uauy C., Shaw P., Moore G. (2017). Exploiting the *ZIP4* homologue within the wheat *Ph1* locus has identified two lines exhibiting homoeologous crossover in wheat-wild relative hybrids. Mol. Breed. New Strateg. Plant Improv..

[188] Baker L., Grewal S., Yang C.-Y., Hubbart-Edwards S., Scholefield D., Ashling S., Burridge A.J., Przewieslik-Allen A.M., Wilkinson P.A., King I.P. (2020). Exploiting the genome of *Thinopyrum elongatum* to expand the gene pool of hexaploid wheat. Theor. Appl. Genet..

[189] Chen P.D., Tsujimoto H., Gill B.S. (1994). Transfer of *Ph(I)* genes promoting homoeologous pairing from *Triticum speltoides* to common wheat. Theor. Appl. Genet..

[190] Chhuneja P., Kaur S., Goel R.K., Aghaee-Sarbarzeh M., Prashar M., Dhaliwal H.S. (2008). Transfer of leaf rust and stripe rust resistance from *Aegilops umbellulata* Zhuk. to bread wheat (*Triticum aestivum* L.). Genet. Resour. Crop Evol..

[191] Aghaee-Sarbarzeh M., Ferrahi M., Singh S., Singh H., Friebe B., Gill B.S., Dhaliwal H.S. (2002). *Ph*-I-induced transfer of leaf and stripe rust-resistance genes from *Aegilops triuncialis* and *Ae. geniculata* to bread wheat. Euphytica.

[192] Aberkane H., Payne T., Kishi M., Smale M., Amri A., Jamora N. (2020). Transferring diversity of goat grass to farmers’ fields through the development of synthetic hexaploid wheat. Food Sec..

[193] Ma’Arup R., Trethowan R.M., Ahmed N.U., Bramley H., Sharp P.J. (2020). Emmer wheat (*Triticum dicoccon* Schrank) improves water use efficiency and yield of hexaploid bread wheat. Plant Sci..

[194] Ullah S., Bramley H., Mahmood T., Trethowan R. (2020). The impact of emmer genetic diversity on grain protein content and test weight of hexaploid wheat under high temperature stress. J. Cereal Sci..

[195] Al Hakimi A., Monneveaux P., Nachit M.M., Braun H., Altay F., Kronstad W.E., Beniwal S.P.S., McNab A. (1997). Direct and indirect selection for drought tolerance in alien tetraploid wheat x durum wheat crosses. Wheat: Prospects for Global Improvement. Developments in Plant Breeding.

[196] Bassi F.M., Brahmi H., Sabraoui A., Amri A., Nsarellah N., Nachit M.M., Al-Abdallat A., Chen M.S., Lazraq A., El Bouhssini M. (2019). Genetic identification of loci for Hessian fly resistance in durum wheat. Mol. Breed..

[197] Liu X., Brown-Guedira G., Hatchett J., Owuoche J., Chen M. (2005). Genetic characterization and molecular mapping of a Hessian fly-resistance gene transferred from *T. turgidum* ssp. *dicoccum* to common wheat. Theor. Appl. Genet..

[198] Rosyara U., Kishii M., Payne T., Sansaloni C.P., Singh R.P., Braun H.J., Dreisigacker S. (2019). Genetic contribution of synthetic hexaploid wheat to CIMMYT’s spring bread wheat breeding germplasm. Sci. Rep..

[199] Kaur A., Chhuneja P., Srivastava P., Singh K., Kaur S. (2021). Evaluation of *Triticum durum–Aegilops tauschii* derived primary synthetics as potential sources of heat stress tolerance for wheat improvement. Plant Genet. Resour..

[200] Börner A., Ogbonnaya F.C., Röder M.S., Rasheed A., Periyannan S., Lagudah E.S., Molnár-Láng M., Ceoloni C., Doležel J. (2015). Aegilops tauschii introgressions in wheat. Alien Introgression in Wheat: Cytogenetics, Molecular Biology, and Genomics.

[201] Gill B.S., Raupp W.J., Sharma H.C., Browder L.E., Hatchett J.H., Harvey T.L., Moseman J.G., Waines J.G. (1986). Resistance in *Aegilops squarrosa* to wheat leaf rust, wheat powdery mildew, greenbug, and Hessian fly. Plant Dis..

[202] Röder M.S., Huang X.-Q., Börner A. (2008). Fine mapping of the region on wheat chromosome 7D controlling grain weight. Funct. Int. Genom..

[203] Maes B., Trethowan R.M., Reynolds M.P., van Ginkel M., Skovmand B. (2001). The influence of glume pubescence on spikelet temperature of wheat under freezing conditions. Austr. J. Plant Pathol..

[204] Reynolds M.P., Saint Pierre C., Saad A.S.I., Vargas M., Condon A.G. (2007). Evaluating potential genetic gains in wheat associated with stress-adaptive trait expression in elite genetic resources under drought and heat stress. Crop Sci..

[205] Szabo-Hever A., Zhang Q., Friesen T.L., Zhong S., Elias E.M., Cai X., Jin Y., Faris J.D., Chao S., Xu S.S. (2018). Genetic diversity and resistance to *Fusarium* head blight in synthetic hexaploid wheat derived from *Aegilops tauschii* and diverse *Triticum turgidum* subspecies. Front. Plant Sci..

[206] Villareal R., Sayre K., Banuelos O., Mujeeb-Kazi A. (2001). Registration of four synthetic hexaploid wheat germplasm lines tolerant to waterlogging. Crop Sci..

[207] Yang J., Sears R.G., Gill B.S., Paulsen G.M. (2002). Growth and senescence characteristics associated with tolerance of wheat-alien amphiploids to high temperature under controlled conditions. Euphytica.

[208] Loughman R., Lagudah E.S., Trottet M., Wilson R.E., Mathews A. (2001). *Septoria nodorum* blotch resistance in *Aegilops tauschii* and its expression in synthetic amphiploids. Austral. J. Agric. Res..

[209] Nicholson P., Rezanoor H.N., Worland A.J. (1993). Chromosomal location of resistance to *Septoria nodorum* in a synthetic hexaploid wheat determined by the study of chromosomal substitution lines in ‘Chinese Spring’ wheat. Plant Breed..

[210] Lage J., Skovmand B., Andersen S.B. (2004). Field evaluation of emmer wheat-derived synthetic hexaploid wheat for resistance to Russian wheat aphid (*Homoptera: Aphididae*). J. Econ. Entomol..

[211] Lage J., Skovmand B., Andersen S.B. (2003). Expression and suppression of resistance to greenbug (*Homoptera: Aphididae*) in synthetic hexaploid wheats derived from *Triticum dicoccum* x *Aegilops tauschii* crosses. J. Econ. Entomol..

[212] King J., Grewal S., Yang C.-Y., Hubbart Edwards S., Scholefield D., Ashling S., Harper J.A., Allen A.M., Edwards K.J., Burridge A.J. (2018). Introgression of *Aegilops speltoides* segments in *Triticum aestivum* and the effect of the gametocidal genes. Ann. Bot..

[213] King J., Grewal S., Yang C.-Y., Hubbart S., Scholefield D., Ashling S., Edwards K.J., Allen A.M., Burridge A., Bloor C. (2017). A step change in the transfer of interspecific variation into wheat from *Amblyopyrum muticum*. Plant Biotechnol. J..

[214] Iefimenko T.S., Fedak Y.G., Antonyuk M.Z., Ternovska T.K. (2015). Microsatellite analysis of chromosomes from the fifth homoeologous group in the introgressive *Triticum aestivum/Amblyopyrum muticum* wheat lines. Cytol. Genet..

[215] King J., Newell C., Grewal S., Hubbart-Edwards S., Yang C.-Y., Scholefield D., Ashling S., Stride A., King I.P. (2019). Development of stable homozygous wheat/*Amblyopyrum muticum* (*Aegilops mutica*) introgression lines and their cytogenetic and molecular characterization. Front. Plant Sci..

[216] Sansaloni C., Franco J., Santos B., Percival-Alwyn L., Singh S., Petroli C., Campos J., Dreher K., Payne T., Marshall D. (2020). Diversity analysis of 80,000 wheat accessions reveals consequences and opportunities of selection footprints. Nat. Commun..

[217] Keilwagen J., Lehnert H., Berner T., Badaeva E., Himmelbach A., Börner A., Kilian B. (2021). Detecting major introgressions in wheat and their putative origin using coverage analysis. Scientific Rep..

[218] Sharma S., Upadhyaya H., Varshney R., Gowda C. (2013). Pre-breeding for diversification of primary gene pool and genetic enhancement of grain legumes. Front. Plant Sci..

[219] Olivera P., Newcomb M., Szabo L.J., Rouse M., Johnson J., Gale S., Luster D.G., Hodson D., Cox J.A., Burgin L. (2015). Phenotypic and genotypic characterization of race TKTTF of *Puccinia graminis* f. sp. *tritici* that caused a wheat stem rust epidemic in southern Ethiopia in 2013–14. Phytopathology®.

[220] Bhattacharya S. (2017). Deadly new wheat disease threatens Europe’s crops. Nature.

[221] Lewis C.M., Persoons A., Bebber D.P., Kigathi R.N., Maintz J., Findlay K., Bueno-Sancho V., Corredor-Moreno P., Harrington S.A., Kangara N. (2018). Potential for re-emergence of wheat stem rust in the United Kingdom. Commun. Biol..

[222] Olivera Firpo P.D., Newcomb M., Flath K., Sommerfeldt-Impe N., Szabo L.J., Carter M., Luster D.G., Jin Y. (2017). Characterization of *Puccinia graminis* f. sp. *tritici* isolates derived from an unusual wheat stem rust outbreak in Germany in 2013. Plant Pathol..

[223] Shamanin V., Salina E., Zelenskiy Y., Kokhmetova A., Patpour M., Hovmøller M., Olivera P., Szabo L., Yue J., Meyer M. Large scale wheat stem rust outbreaks in Western Siberia/Northern Kazakhstan in 2015-2017. Proceedings of the Borlaug Global Rust Initiative: Technical Workshop.

[224] Kildea S., Sheppard L., Cucak M., Hutton F. (2021). Detection of virulence to *Septoria tritici* blotch (STB) resistance conferred by the winter wheat cultivar Cougar in the Irish *Zymoseptoria tritici* population and potential implications for STB control. Plant Pathol..

[225] Wang X., Liu Y., Chen L., Zhao D., Wang X., Zhang Z. (2013). Wheat resistome in response to barley yellow dwarf virus infection. Funct. Int. Genom..

[226] Hussain B., Akpınar B.A., Alaux M., Algharib A.M., Sehgal D., Ali Z., Appels R., Aradottir G.I., Batley J., Bellec A. (2021). Wheat genomics and breeding: Bridging the gap. AgriRxiv.

[227] Hoffmann B., Burucs Z. (2005). Adaptation of wheat (*Triticum aestivum* L.) genotypes and related species to water deficiency. Cereal Res. Commun..

[228] Rajaram S. (2001). Prospects and promise of wheat breeding in the 21^st^ century. Euphytica.

[229] Sallam A., Alqudah A.M., Dawood M.F.A., Baenziger P.S., Börner A. (2019). Drought stress tolerance in wheat and barley: Advances in physiology, breeding and genetics research. Int. J. Mol. Sci..

[230] Damania A.B., Altunji H., Dhaliwal H.S. (1992). Evaluation of Aegilops spp. for Drought and Frost Tolerance.

[231] Jaradat A.A. (2011). Ecogeography, genetic diversity, and breeding value of wild emmer wheat (*Triticum dicoccoides* Körn. ex Asch. & Graebn.) Thell. Austral. J. Agric. Res..

[232] Nevo E., Chen G. (2010). Drought and salt tolerances in wild relatives for wheat and barley improvement. Plant Cell Environ..

[233] Dixon J., Braun H.-J., Kosina P., Crouch J.H. (2009). Wheat Facts and Futures.

[234] Zaharieva M., Ayana N., Hakimi A., Misra S., Monneveux P. (2010). Cultivated emmer wheat (*Triticum dicoccon* Schrank), an old crop with promising future: A review. Genet. Resour. Crop Evol..

[235] Zaharieva M., Monneveux P. (2014). Cultivated einkorn wheat (*Triticum monococcum* L. subsp. *monococcum*): The long life of a founder crop of agriculture. Genet. Resour. Crop Evol..

[236] Oliver R.E., Cai X., Friesen T.L., Halley S., Stack R.W., Xu S.S. (2008). Evaluation of *Fusarium* head blight resistance in tetraploid wheat (*Triticum turgidum* L.). Crop Sci..

[237] Saleh M.M. (2020). Stress breeding of neglected tetraploid primitive wheat (*Triticum dicoccum, Triticum carthlicum* and *Triticum polonicum*). Curr. Bot..

[238] Bencze S., Makádi M., Aranyos T.J., Földi M., Hertelendy P., Mikó P., Bosi S., Negri L., Drexler D. (2020). Re-introduction of ancient wheat cultivars into organic agriculture—Emmer and einkorn cultivation experiences under marginal conditions. Sustainability.

[239] Bonafaccia G., Galli V., Francisci R., Mair V., Skrabanja V., Kreft I. (2000). Characteristics of spelt wheat products and nutritional value of spelt wheat-based bread. Food Chem..

[240] Konvalina P., Stehno Z., Capouchová I., Moudrý jr. J., Jůza M., Moudrý J. (2010). Emmer wheat using and growing in the Czech Republic. Lucr. Ştiinţifice Ser. Agron..

[241] Lacko-Bartošová M., Čurná V., Lacko-Bartošová L. (2015). Emmer—Ancient wheat suitable for ecological farming. Res. J. Agric. Sci..

[242] Stagnari F., Codianni P., Pisante M. (2008). Agronomic and kernel quality of ancient wheats grown in central and southern Italy. Cereal Res. Commun..

[243] Escarnot E., Jacquemin J.M., Agneessens R., Paquot M. (2012). Comparative study of the content and profiles of macronutrients in spelt and wheat, a review. Biotech. Agron. Soc. Environ..

[244] Yenagi N., Hanchinal R.R., Patil C.S., Koppikar V., Halagi M. (2001). Glycemic and lipidemic response to dicoccum wheat (*Triticum dicoccum*) in the diet of diabetic patients. Int. J. Diabetes Dev. Ctries..

[245] Hidalgo A., Brandolini A. (2014). Nutritional properties of einkorn wheat (*Triticum monococcum* L.). J. Sci. Food Agric..

[246] Wiwart M., Suchowilska E., Kandler W., Sulyok M., Groenwald P., Krska R. (2013). Can Polish wheat (*Triticum polonicum* L.) be an interesting gene source for breeding wheat cultivars with increased resistance to *Fusarium* head blight?. Genet. Resour. Crop Evol..

[247] Cooper R. (2015). Re-discovering ancient wheat varieties as functional foods. J. Tradit. Complement. Med..

[248] Stallknecht G.F., Gilbertson K.M., Ranney J.E., Janick J. (1996). Alternative wheat cereals as food grains: Einkorn, emmer, spelt, kamut, and triticale. Progress in New Crops.

[249] Bond A. (1989). Discovering einkorn in Haute Provence, France. Cerealist.

[250] Galterio G., Codianni P., Giusti A.M., Pezzarossa B., Cannella C. (2003). Assessment of the agronomic and technological characteristics of *Triticum turgidum* ssp. *dicoccum* Schrank and *T*. *spelta* L.. Nahrung.

[251] Keskin Şan S., Özbek Ö., Eser V., Göçmen Taşkin B. (2015). Polymorphism in seed endosperm proteins (gliadins and glutenins) of Turkish cultivated einkorn wheat [*Triticum monococcum* ssp. *monococcum*] landraces. Cereal Res. Commun..

[252] Cakmak I., Torun A., Millet E., Feldman M., Fahima T., Korol A., Nevo E., Braun H.J., Özkan H. (2004). *Triticum dicoccoides*: An important genetic resource for increasing zinc and iron concentration in modern cultivated wheat. Soil Sci. Plant Nut..

[253] García A.B., Castellano L., Guzmán C., Alvarez J.B. (2021). Potential use of wild einkorn wheat for wheat grain quality improvement: Evaluation and characterization of *Glu-1*, Wx and *Ha* loci. Agronomy.

[254] Liu J., Huang L., Li T., Liu Y., Yan Z., Tang G., Zheng Y., Liu D., Wu B. (2021). Genome-wide association study for grain micronutrient concentrations in wheat advanced lines derived from wild emmer. Front. Plant Sci..

[255] Erba D., Hidalgo A., Bresciani J., Brandolini A. (2011). Environmental and genotypic influences on trace element and mineral concentrations in whole meal flour of einkorn (*Triticum monococcum* L. subsp. *monococcum*). J. Cereal Sci..

[256] Özkan H., Brandolini A., Torun A., Altintas S., Eker S., Kilian B., Braun H.J., Salamini F., Cakmak I., Buck H.T., Nisi J.E., Salomon N. (2007). Natural variation and identification of microelements content in seeds of einkorn wheat *(Triticum monococcum*). Wheat Production in Stressed Environments.

[257] Chhuneja P., Dhaliwal H.S., Bains N.S., Singh K. (2006). *Aegilops kotschyi* and *Aegilops tauschii* as sources for higher levels of grain iron and zinc. Plant Breed..

[258] Kumar A., Kapoor P., Chunduri V., Sharma S., Garg M. (2019). Potential of *Aegilops* sp. for improvement of grain processing and nutritional quality in wheat (*Triticum aestivum*). Front. Plant Sci..

[259] Rawat N., Tiwari V.K., Singh N., Randhawa G.S., Singh K., Chhuneja P., Dhaliwal H.S. (2009). Evaluation and utilization of *Aegilops* and wild *Triticum* species for enhancing iron and zinc content in wheat. Genet. Resour. Crop Evol..

[260] Leonova I.N., Budashkina E.B., Kalinina N.P., Röder M.S., Börner A., Salina E.A. (2011). *Triticum aestivum-Triticum timopheevii* introgression lines as a source of pathogen resistance genes. Czech J. Genet. Breed..

[261] EiB. https://excellenceinbreeding.org/.

[262] Jakob S.S., Rödder D., Engler J.O., Shaaf S., Özkan H., Blattner F.R., Kilian B. (2014). Evolutionary history of wild barley (*Hordeum vulgare* subsp. *spontaneum*) analyzed using multilocus sequence data and paleodistribution modeling. Gen. Biol. Evol..

[263] Singh N., Wu S., Raupp W.J., Sehgal S., Arora S., Tiwari V., Vikram P., Singh S., Chhuneja P., Gill B.S. (2019). Efficient curation of genebanks using next generation sequencing reveals substantial duplication of germplasm accessions. Sci. Rep..

[264] Brown A.H.D. (1989). Core collections: A practical approach to genetic resources management. Genome.

[265] Upadhyaya H.D., Ortiz R. (2001). A mini core subset for capturing diversity and promoting utilization of chickpea genetic resources in crop improvement. Theor. Appl. Genet..

[266] Upadhyaya H.D., Dwivedi S.L., Baum M., Varshney R.K., Udupa S.M., Gowda C.L.L., Hoisington D., Singh S. (2008). Genetic structure, diversity, and allelic richness in composite collection and reference set in chickpea (*Cicer arietinum* L.). BMC Plant Biol..

[267] Glaszmann J.C., Kilian B., Upadhyaya H.D., Varshney R.K. (2010). Accessing genetic diversity for crop improvement. Curr. Opin. Plant Biol..

[268] Khazaei H., Street K., Bari A., Mackay M., Stoddard F.L. (2013). The FIGS (Focused Identification of Germplasm Strategy) approach identifies traits related to drought adaptation in *Vicia faba* genetic resources. PLoS ONE.

[269] Lande R., Thompson R. (1990). Efficiency of marker-assisted selection in the improvement of quantitative traits. Genetics.

[270] Chaudhary H.K., Sethi G.S., Singh S., Pratap A., Sharma S. (2005). Efficient haploid induction in wheat by using pollen of *Imperata cylindrica*. Plant Breed..

[271] Ghosh S., Watson A., Gonzalez-Navarro O.E., Ramirez-Gonzalez R.H., Yanes L., Mendoza-Suárez M., Simmonds J., Wells R., Rayner T., Green P. (2018). Speed breeding in growth chambers and glasshouses for crop breeding and model plant research. Nat. Protoc..

[272] Sharma S., Sethi G.S., Chaudhary H.K. (2005). Influence of winter and spring wheat genetic backgrounds on haploid induction parameters and trait correlations in the wheat × maize system. Euphytica.

[273] Arora S., Steuernagel B., Gaurav K., Chandramohan S., Long Y., Matny O., Johnson R., Enk J., Periyannan S., Singh N. (2019). Resistance gene cloning from a wild crop relative by sequence capture and association genetics. Nat. Biotechnol..

[274] Bai G., Kolb F.L., Shaner G., Domier L.L. (1999). Amplified fragment length polymorphism markers linked to a major quantitative trait locus controlling scab resistance in wheat. Phytopathology®.

[275] Rawat N., Pumphrey M.O., Liu S., Zhang X., Tiwari V.K., Ando K., Trick H.N., Bockus W.W., Akhunov E., Anderson J.A. (2016). Wheat *Fhb1* encodes a chimeric lectin with agglutinin domains and a pore-forming toxin-like domain conferring resistance to *Fusarium* head blight. Nat. Genet..

[276] Waldron B., Moreno-Sevilla B., Anderson J., Stack R., Frohberg R. (1999). RFLP mapping of QTL for *Fusarium* head blight resistance in wheat. Crop Sci..

[277] Bernardo R. (2009). Genomewide selection for rapid introgression of exotic germplasm in maize. Crop Sci..

[278] Meuwissen T.H., Hayes B.J., Goddard M.E. (2001). Prediction of total genetic value using genome-wide dense marker maps. Genetics.

[279] VanRaden P.M. (2008). Efficient methods to compute genomic predictions. J. Dairy Sci..

[280] Arbelaez J.D., Moreno L.T., Singh N., Tung C.-W., Maron L.G., Ospina Y., Martinez C.P., Grenier C., Lorieux M., McCouch S. (2015). Development and GBS-genotyping of introgression lines (ILs) using two wild species of rice, *O. meridionalis* and *O. rufipogon*, in a common recurrent parent, *O. sativa* cv. Curinga. Mol. Breed..

[281] Nyine M., Adhikari E., Clinesmith M., Jordan K.W., Fritz A.K., Akhunov E. (2020). Genomic patterns of introgression in interspecific populations created by crossing wheat with its wild relative. G3 Genes|Genomes|Genet..

[282] Schnell F.W., Utz H.F. (1976). F1 Leistung und Elternwahl in der Züchtung von Selbstbefruchtern. Bericht über die Arbeitstagung 1975 der Arbeitsgemeinschaft der Saatgutleiter.

[283] Becker H. (2011). Pflanzenzüchtung.

[284] Endelman J.B., Atlin G.N., Beyene Y., Semagn K., Zhang X., Sorrells M.E., Jannink J.-L. (2014). Optimal design of preliminary yield trials with genome-wide markers. Crop Sci..

[285] Holland J.B., Nyquist W.E., Cervantes-Martínez C.T., Janick J. (2003). Estimating and interpreting heritability for plant breeding: An update. Plant Breeding Reviews.

[286] Piepho H.-P., Möhring J. (2007). Computing heritability and selection response from unbalanced plant breeding trials. Genetics.

[287] Allier A., Moreau L., Charcosset A., Teyssèdre S., Lehermeier C. (2019). Usefulness criterion and post-selection parental contributions in multi-parental crosses: Application to polygenic trait introgression. G3.

[288] Lehermeier C., Teyssèdre S., Schön C.C. (2017). Genetic gain increases by applying the usefulness criterion with improved variance prediction in selection of crosses. Genetics.

[289] Mrode R., Brotherstone S., White I., Swanson G., Coffey M., Jones H., Thompson R. (2005). Random regression model for the genetic evaluation of production traits of dairy cattle in the UK. Interbull Bull..

[290] Bernardo R. (2014). Genomewide selection of parental inbreds: Classes of loci and virtual biparental populations. Crop Sci..

[291] Mohammadi M., Tiede T., Smith K.P. (2015). PopVar: A genome-wide procedure for predicting genetic variance and correlated response in biparental breeding populations. Crop Sci..

[292] Gianola D., Fernando R.L., Stella A. (2006). Genomic-assisted prediction of genetic value with semiparametric procedures. Genetics.

[293] Gianola D., van Kaam J.B.C.H.M. (2008). Reproducing kernel Hilbert spaces regression methods for genomic assisted prediction of quantitative traits. Genetics.

[294] Jiang Y., Reif J.C. (2015). Modeling epistasis in genomic selection. Genetics.

[295] Mullis M.N., Matsui T., Schell R., Foree R., Ehrenreich I.M. (2018). The complex underpinnings of genetic background effects. Nat. Commun..

[296] Merchuk-Ovnat L., Fahima T., Ephrath J.E., Krugman T., Saranga Y. (2017). Ancestral QTL alleles from wild emmer wheat enhance root development under drought in modern wheat. Front. Plant Sci..

